# Beyond bacteria: Reconstructing microorganism connections and deciphering the predicted mutualisms in mammalian gut metagenomes

**DOI:** 10.1002/ece3.9829

**Published:** 2023-02-22

**Authors:** Qinlong Dai, Jingjing Ding, Xinyuan Cui, Yudong Zhu, Hua Chen, Lifeng Zhu

**Affiliations:** ^1^ Sichuan Liziping National Natural Reserve Shimian China; ^2^ Jiangsu Academy of Forest Nanjing China; ^3^ College of Life Science Nanjing Normal University Nanjing China; ^4^ Mingke Biotechnology (Hangzhou) Co., Ltd. Hangzhou China; ^5^ College of Pharmacy Nanjing University of Chinese Medicine Nanjing China

**Keywords:** mammal metagenomes, microorganisms, putative interaction

## Abstract

Numerous gut microbial studies have focused on bacteria. However, archaea, viruses, fungi, protists, and nematodes are also regular residents of the gut ecosystem. Little is known about the composition and potential interactions among these six kingdoms in the same samples. Here, we unraveled the complex connection among them using approximately 123 gut metagenomes from 42 mammalian species (including carnivores, omnivores, and herbivores). We observed high variation in bacterial and fungal families and relatively low variation in archaea, viruses, protists, and nematodes. We found that some fungi in the mammalian intestine might come from environmental sources (e.g., soil and dietary plants), and some might be native to the intestine (e.g., the occurrence of *Neocallimastigomycetes*). The *Methanobacteriaceae* and *Plasmodiidae* families (archaea and protozoa, respectively) were predominant in these metagenomes, whereas *Onchocercidae* and *Trichuridae* were the two most common nematodes, and *Siphoviridae* and *Myoviridae* the two most common virus families in these mammalian gut metagenomes. Interestingly, most of the pairwise co‐occurrence patterns were significantly positive among these six kingdoms, and significantly negative networks mainly occurred between fungi and prokaryotes (both bacteria and archaea). Our study revealed some inconvenient characteristics in the mammalian gut microorganism ecosystem: (1) the community formed by members of the analyzed kingdoms reflects the life history of the host and the potential threat posed by pathogenic protists and nematodes in mammals; and (2) the networks suggest the existence of predicted mutualism among members of these six kingdoms and of the predicted competition, mainly among fungi and other kingdoms.

## Graphical abstract

Here, we unraveled the complex connection among them using approximately 123 gut metagenomes from 42 mammalian species (including carnivores, omnivores, and herbivores). Our study revealed the existence of predicted mutualism among members of these six kingdoms (e.g., archaea, bacteria, viruses, fungi, protists, and nematodes) and of the predicted competition were mainly among fungi and other kingdoms.

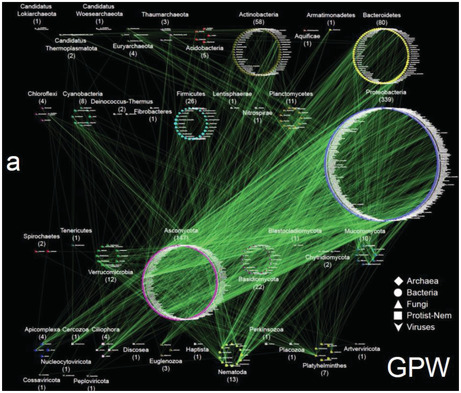

## 1 INTRODUCTION

The gut microbiome plays an important role in host health and diseases, and many studies have associated it with bacteria (prokaryotes), which are the main microorganisms in the digestive system (Huang et al., 2022; Ley et al., 2008a, 2008b; Wei et al., 2019; Youngblut et al., 2019). Other microorganisms in the gut include archaea (prokaryotes), viruses, and eukaryotes, such as fungi, protists, and nematodes (Richard & Sokol, 2019). The balance in the symbiotic relationships that exist among the members of these kingdoms is a key factor in maintaining the health of the host. Until now, most gut microbial research has focused on the interaction between the host and its gut bacteria. However, the putative networks existing among the members of the previously mentioned kingdoms have been poorly explored. The development of metagenomic sequencing has pushed forward microbial research, and an increasing number of studies have discussed the function of gut microorganisms, especially that of bacteria (Qin et al., 2010; Streit & Schmitz, 2004; Thomas et al., 2012; Zhu et al., 2011). Interestingly, metagenomes enable researchers to simultaneously study the entire collection of genomes from a mixed population of microorganisms. This is invaluable for understanding animal gut ecosystems. Therefore, we can simultaneously investigate the co‐occurrence patterns among archaea, bacteria, viruses, fungi, protists, and nematodes. This is a neglected characteristic in studies on gut microorganisms.

Network‐based approaches have proven valuable for exploring complex interactions in systems biology and are also widely used in gut microbial studies (Layeghifard et al., 2017). We aimed at deciphering the existing connections among gut microorganisms using mammalian gut metagenomes. To begin, we chose 123 gut metagenomes from 42 mammalian species (including carnivores, omnivores, and herbivores); most of these metagenomes (approximately 104) came from our laboratory; therefore, they were obtained using the same sequencing platform to decrease the sequencing bias (Appendix S1). The details on the origin of the 123 metagenomes are as follows: 52 from giant pandas (9 from the Qinling Mountains [GPQIN, wild] (Wu et al., 2017), 7 from the Qionglai Mountains [GPQIO, wild] (Guo et al., 2019), 19 from the Xiaoxiangling Mountains [GPXXL, wild] (Yao et al., 2021; Zhu, Yang, et al., 2018), 7 from the Chengdu Breeding Center [GPCD, captive] (Zhang et al., 2018), 10 from the Yaan Research Base of the Wolong Research Center [GPYA, captive]) (Guo et al., 2019), 6 from red pandas from the Xiaoxiangling Mountains (RP, wild; Zhu, Yang, et al., 2018), 19 from meat‐eating carnivorans (CA) (Zhu, Wu, et al., 2018), 10 from omnivorous carnivorans (OC) (Guo et al., 2018; Zhu, Wu, et al., 2018), 12 from herbivores (HE) (Zhu, Wu, et al., 2018), and 24 from Yunnan snub‐nosed monkeys (YSNM, wild) (Xia et al., 2022). We investigated the gut microorganism composition regarding the previously mentioned kingdoms and compared their beta diversities. Finally, we investigated the interactions among such microorganisms.

## 2 RESULTS AND DISCUSSION

### 2.1 Family‐level identification among six kingdoms across the different mammal groups

**FIGURE 1:**
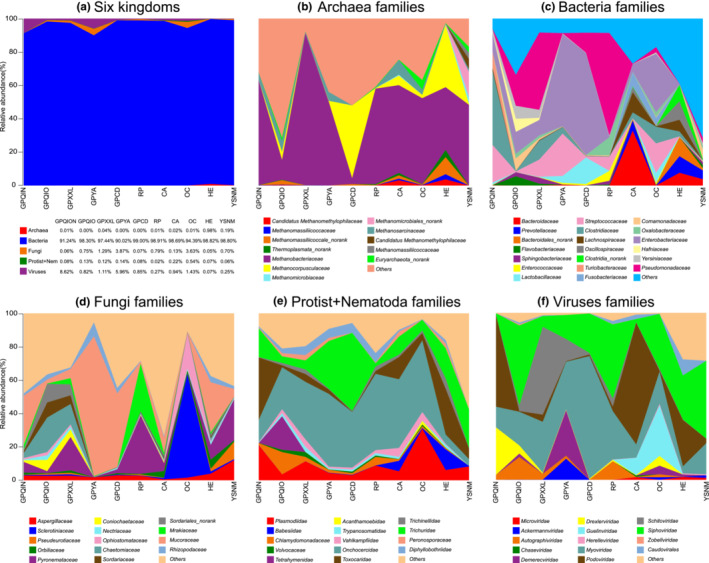
Relative abundance of microorganism from six different kingdoms in the gut of different mammals. (a) All six kingdoms; (b) archaeal families; (c) bacterial families; (d) fungal families; (e) protist and nematode families (combined); (f) virus families. The 123 metagenomes used in these analyses belonged to 52 giant pandas (9 from the Qinling Mountains [GPQIN, wild] (Wu et al., 2017), 7 from the Qionglai Mountains [GPQIO, wild] (Guo et al., 2019), 19 from the Xiaoxiangling Mountains [GPXXL, wild] (Yao et al., 2021; Zhu, Wu, et al., 2018), 7 from the Chengdu Breeding Center [GPCD, captive] (Zhang et al., 2018), and 10 from the Yaan research base of the Wolong Research Center [GPYA, captive]) (Guo et al., 2019); 6 red pandas from the Xiaoxiangling Mountains (RP, wild) (Zhu, Wu, et al., 2018); 19 meat‐eating carnivorans (CA) (Zhu, Yang, et al., 2018); 10 omnivorous carnivorans (OC) (Guo et al., 2018; Zhu, Wu, et al., 2018); 12 herbivores (HE) (Zhu, Wu, et al., 2018); and 24 Yunnan snub‐nosed monkeys (YSNM, wild) (Xia et al., 2022).

We retained the reads from the previously mentioned kingdoms (archaea, bacteria, viruses, fungi, protists, and nematodes) in the 123 metagenomes analyzed; we observed high variation in the bacterial and fungal families and relatively low variation in archaea, viruses, and protists‐nematodes (Figure 1). In the metagenomes, most of the reads belonged to the bacterial kingdom (>90%, Figure 1c). As previously identified, the relative abundance of *Pseudomonadaceae* is high in wild giant panda (GPXXL and GPQIO, but not GPQIN) and RP populations, and this bacterial family is putatively involved in secondary plant metabolites (e.g., dietary cyanide compounds) detoxification (Hu et al., 2021; Wang et al., 2021; Zhu, Yang, et al., 2018). Captive giant pandas and OC harbor a high proportion of *Enterobacteriaceae* (Figure 1c), most probably due to their living environments (Huang et al., 2021; Zhu, Wu, et al., 2011, 2018). HE and YSNM harbor a high proportion of *Prevotellaceae* (Figure 1c), which might be related to the high proportion of carbohydrates in their diet (Xia et al., 2022; Zhu, Wu, et al., 2018).

Regarding fungi, the proportion of *Mucoraceae* was high in four mammal groups (three captive groups: GPYA, GPCD, and HE, and one wild group: GPQIN; Figure 1d). *Mucoraceae* are saprophytic fungi; many common molds that are destructive to food products belong to this family (Cavalcanti & Trufem, 2008). We speculated that dietary plants (e.g., bamboo) might be the source of these fungi. Wild mammals GPXXL, RP, and YSNM groups had a relatively high proportion of *Pyronemataceae* compared with the captive mammal groups GPYA, GPCD, CA, and OC (Figure 1d). The wild RP and GPXXL, which live in the same region, have a similar abundance pattern: a relatively high proportion of fungi from the Mrakiaceae family. *Pyronemataceae* comprises saprobic, ectomycorrhizal, bryosymbiotic, and parasitic species that occur in a broad range of habitats (in soil, burnt ground, debris, wood, and dung and inside living bryophytes, plants, and lichens; Hansen et al., 2013). For example, the main diet of the YSNM is lichen. Thus, a high proportion of *Pyronemataceae* in wild herbivorous carnivoran (giant panda and RP) and monkey feces may be associated with dietary plants. OC harbored a high proportion of *Sclerotiniaceae* and *Ophiostomataceae* (Figure 1d). Sclerotiniaceae is a family of fungi in the order *Helotiales*, and many species in this family are plant pathogens (Whetzel, 1945). The *Ophiostomataceae* family has a widespread distribution, and its members are pathogens of both coniferous and deciduous trees (Solheim, 1986). Their presence in OC mammals might be related to dietary plants as a source of fungi. CAs have different dominant microorganism families compared with other mammal groups. Therefore, bacterial and fungal diversities are affected at the family level by captivity (i.e., the living environment), given that captive and wild pandas harbor different core bacteria and fungi; by dietary plants, given that typical plant pathogenic fungi may be found in the feces of some of the mammals under study; and by the host itself, given that CA and OC harbor different bacterial and fungal families. Anaerobic gut fungi (e.g., class *Neocallimastigomycetes*) are important members of the gut microbiome of herbivores (Swift et al., 2021). We also found that some reads (mainly from the metagenomes from giant pandas, OC, HE, and YSNM) matched *Neocallimastigomycetes* (Appendix S1); most of them putatively belonged to *Neocallimastix californiae* and *Anaeromyces robustus*, which live in the native rumen environment and can degrade cellulose (Henske et al., 2018; Swift et al., 2019). The relative abundance of the putative *Neocallimastigomycetes* in HE or YSNM was significantly higher than that of other mammal groups in this study (Appendix S1).

We do not know whether the fungi identified in this study by metagenomic methods can live in the mammalian intestine because we used genomic DNA extracted from feces and not culture methods. However, indirect evidence suggested that some of the fungi found in the mammalian intestine might come from environmental sources (e.g., soil and dietary plants), whereas some might be native to the intestine (e.g., putative *Neocallimastigomycetes*).

**FIGURE 2:**
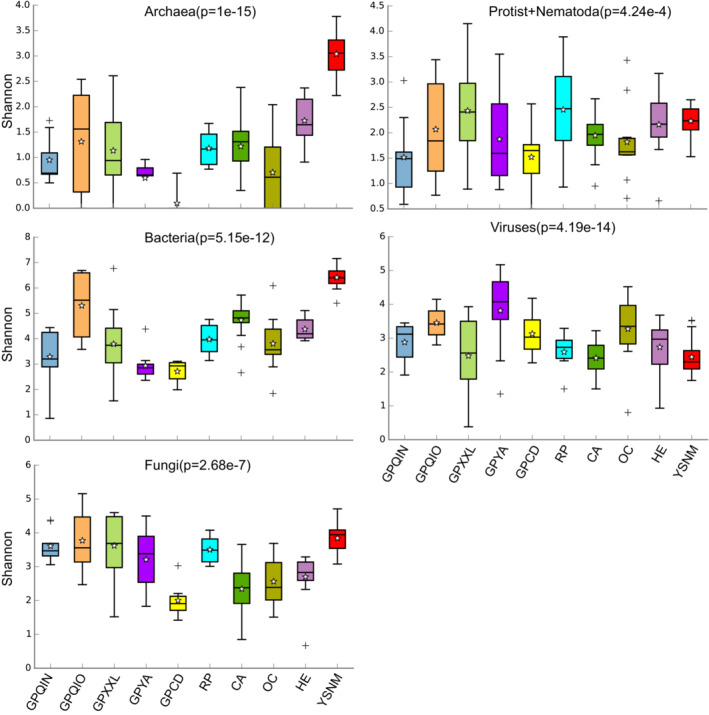
Shannon index of each microorganism kingdom among these 10 mammal groups.

Moreover, we observed that the microbial composition of the studied mammal groups was relatively conserved at the family level for archaea, protists, and nematodes compared with that for bacteria and fungi, whereas their alpha diversity was highly different. Archaea *Methanobacteriaceae* (with a predominance of the genus *Methanobrevibacter*) was the dominant family across the mammal groups under study (except for GPCD), and the relative abundance of *Methanocorpusculaceae* (with a predominance of the genus *Methanocorpusculum*) was high in HE (Figure 1b, Appendix S1). The relative abundance of the putative *Neocallimastigomycetes* in HE was nearly significantly higher than that of other mammal groups in this study (Appendix S1). The species within *Methanobrevibacter* are strictly anaerobic archaea that produce methane, mostly through the reduction of carbon dioxide via hydrogen. Most species live in the intestines of animals, such as termites (Leadbetter & Breznak, 1996) and humans (Samuel et al., 2007). Putatively, *Methanobrevibacter smithii*, *Methanobrevibacter olleyae*, and *Methanobrevibacter* sp. were identified in the samples, confirming the results of previous studies (Leadbetter & Breznak, 1996; Samuel et al., 2007). Among the mammal groups, herbivores (including HE and YSNM) had the high alpha diversity (Shannon index), especially in Archaea and Bacteria (Figure 2), which was similar to the previous studies (Ley et al., 2008a, 2008b; Zhu, Wu, et al., 2018). The complexity of the dietary diversity might partially result in this pattern.

Regarding the Protista and Nematoda kingdoms, *Protozoa Plasmodiidae* (mainly represented by the genus *Plasmodium*) was the domain family in our metagenomes (Figure 1e). *Plasmodium* is a parasite that infects humans, primates, rodents, birds, and reptiles and is distributed worldwide (Aravind et al., 2003). *Onchocercidae* (mainly represented by the genera *Brugia*, *Onchocerca*, and *Wuchereri*) and Trichuridae (mainly represented by *Trichuris*) are the two most common Nematoda families in our mammalian metagenomes. Among *Trichuris*, most reads were putatively mapped to *Trichuris trichiura*, which is one of the main parasites in humans (especially children) (Stephenson et al., 2000). Regarding *Brugia*, some reads were putatively mapped to *Brugia malayi*, a human filarial parasite that threatens human health (Ghedin et al., 2007). Although we cannot confirm that the previously mentioned reads in our metagenomes belong to *Plasmodium*, *T*. *trichiura*, and *B*. *malayi*, we can conclude that these parasite species are our reads' closest relatives according to the current NCBI micro‐NR database. Therefore, regardless of the living conditions (captive or wild habitat) or the host species, most of the mammals included in this study face the potential threat of being infected by parasitic protists and nematodes.

In addition, *Siphoviridae* (including *Jerseyvirus*, *Kagunavirus*, *Dhillonvirus*, *Lambdavirus*, and *Seuratvirus*) and *Myoviridae* (including *Dhakavirus*, *Asteriusvirus*, *Gaprivervirus*, *Kanagawavirus*, *Krischvirus*, *Phapecoctavirus*, and *Felixounavirus*) are the two most common bacterial virus (phages) families in our mammal gut metagenomes (Figure 1f). *Siphoviridae* and *Myoviridae* can infect strains of *Enterobacteriaceae*, *Pseudomonadaceae*, and *Lactobacillaceae* (Brüssow & Desiere, 2001; Lavigne et al., 2009). Despite the high variation in the bacterial families present in the mammal groups included in this study, large broad‐spectrum polyvalent infections (including both gram‐negative and gram‐positive bacteria) resulted in a conserved bacteriophage pattern at the family level in our metagenomes. However, the virus composition partially shows the specific features of the mammal group. For example, the relative abundance of *Ackermannviridae* and *Demerecviridae* was high in GPYA, while OC harbored a high proportion of *Guelinviridae*, GPXXL harbored a high proportion of *Schitoviridae*, and CA harbored the highest proportion of *Podoviridae* (Figure 1f).

**FIGURE 3:**
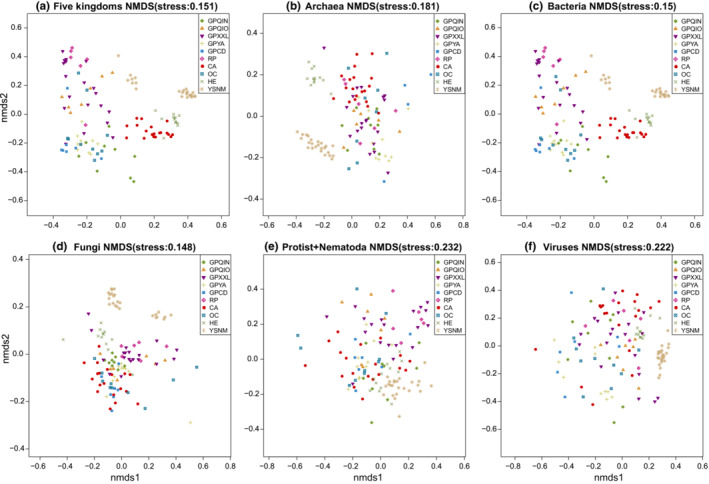
NMDS ordination analysis among different mammal groups based on Bray–Curtis distance matrices using the TPM of six microorganism kingdoms. NMDS: (a) using all kingdoms at the species level; (b) using archaea at the species level; (c) using bacteria at the species level; (d) using fungi at the species level; (e) using protists and nematodes at the species level (in combination); (f) using viruses at the species level.

We speculated that host‐specific patterns of microorganisms from the six kingdoms included in this study could be determined at the species or strain level. Results from NMDS analyses using Bray–Curtis distance of the putative species‐level abundance support our hypothesis that mammal groups have significantly different mean abundance dissimilarities regarding microorganisms from the six kingdoms under study (Figure 3a, Figure 2f; Adonis test: *p* = .001). The highest variation in distances was observed among bacteria, whereas the lowest was observed among viruses in all mammal groups (Figure 2; *R* ^2^ of bacteria: 43.7%; *R* ^2^ of fungi: 30.1%; *R* ^2^ of protists‐nematodes: 29.5%; *R* ^2^ of archaea: 27.7%; *R* ^2^ of virus: 21.6%). Beta analyses at the putative species level further confirmed the relatively high variations in bacterial and fungal kingdoms compared with those in archaea, protists‐nematodes, and viruses.

Overall, at the family level, core microorganisms of the six kingdoms under study reflected the effects of the life history or ecological niches of the host. The variation in the abundance of bacteria and fungi among mammal groups indicates the potential effects of diet, host phylogeny, and living conditions. The other four kingdoms (i.e., archaea, viruses, protists, and nematodes) show three kinds of “broad‐spectrum polyvalent” characteristics. Archaea in the mammalian intestine are strictly anaerobic and reduce carbon dioxide via the hydrogen produced by the fermentation of carbohydrates by bacteria. Phages in the mammalian gut can infect both gram‐negative and gram‐positive bacteria. These characteristics mimic the variation in the abundance of specific bacterial groups among mammals. Furthermore, although the parasitic protist and nematode species found in mammals might be different, the close phylogenetic position at the family level and potential capability for cross‐species infection among different hosts lead to similar core parasite families among the mammal groups, regardless of their life history differences. Parasites are more dependent on the host than symbiotic bacteria. Thus, the “broad‐spectrum polyvalent” characteristics described here may have led to “convergence” at the family level in archaea, viruses, protists, and nematodes. However, the potential interactions among the six microorganism kingdoms require further exploration.

### 2.2 Co‐occurrence networks among the six microorganism kingdoms reflecting the predicted competition

**FIGURE 4:**
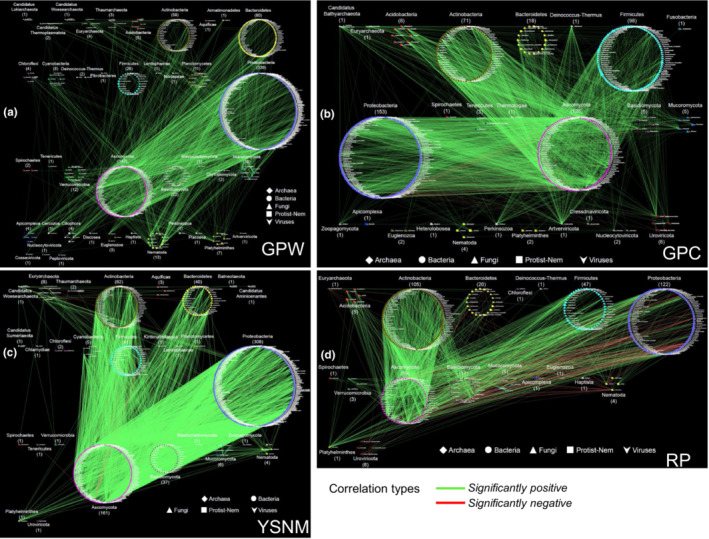
Complex interactions among the six microorganism kingdoms studied in specific mammal populations. (a) GPW group (combining GPQIN, GPQIO, and GPXXL; Spearman: *r* ≥ .8, *p* ≤ .01); (b) GPC group (combining GPYA and GPCD; Spearman: *r* ≥ .8, *p* ≤ .01); (c) YSNM group (Spearman: r ≥ .8, p ≤ .01); (d) RP (Spearman: *r* ≥ .9, *p* ≤ .01). Green lines represent significantly positive correlations. Red lines represent significantly negative correlations. Each point represents a single genus from the indicated family belonging to one of the six kingdoms analyzed (except for viruses for which families are represented).

**FIGURE 5:**
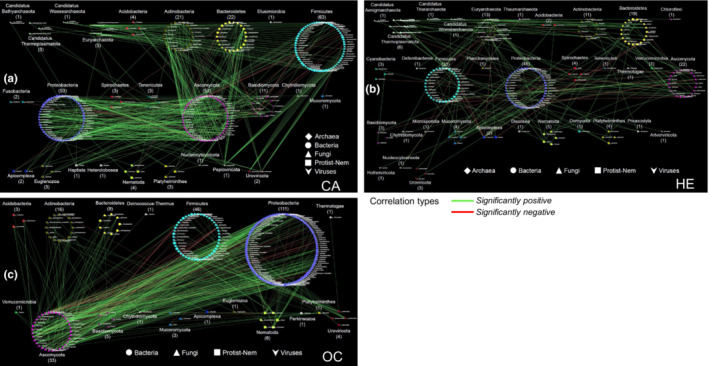
Complex interactions among the six microorganism kingdoms studied in specific mammal populations. (a) CA group (Spearman *r* ≥ .8, *p* ≤ .01); (b) HE group (Spearman *r* ≥ .9, *p* ≤ .01); (c) OC (Spearman *r* ≥ .9, *p* ≤ .01). Green lines represent significantly positive correlations. Red lines represent significantly negative correlations. Each point represents a single genus from the indicated family belonging to one of the six kingdoms analyzed (except for viruses for which families are represented).

We next focused on transkingdom interactions among the six kingdoms in the mammal metagenomes through the lens of specific life histories or ecological niches. Interestingly, most of the pairwise co‐occurrence patterns were significantly positive among the kingdoms, and significantly negative networks mainly occurred between fungi and prokaryotes (both bacteria and archaea; Appendix S1). Most of the interactions between archaea genera and members of the other kingdoms (e.g., bacteria) were significantly positive among mammal groups, including captive giant pandas (GPC; combined GPYA and GPCD), wild giant pandas (GPW; combined GPQIN, GPQIO, and GPXXL), YSNM, CA, and HE (Figures 4 and 5; Appendix S1). Virus families displayed a similar pattern (Appendix S1). These findings indicate predicted mutualisms between bacteria and archaea or viruses. Archaea (e.g., *Methanobacteriales*, *M*. *smithii*, and *Methanosphaera stadtmanae*) are naturally occurring components of the animal gut microbiota, and they participate in functions such as methanogenesis, transformation of heavy metals, and trimethylamine metabolism (Brugère et al., 2014; Leadbetter & Breznak, 1996; Samuel et al., 2007). Thus, the complex network across different mammalian groups investigates the predicted mutualism between archaea and bacteria in the mammalian gut ecosystem. Bacteriophages are the predominant viruses in the human gut microbiome, and they shape the microbial composition and drive bacterial diversity and nutrient turnover through continuous cycles of predation and coevolution (Sutton & Hill, 2019). Maintaining a dynamic balance between bacteriophages and bacteria is beneficial. Here, we also revealed that DNA viruses (e.g., phages) show some kind of interdependence with bacteria across different mammalian groups. Therefore, in this study, we provide evidence of potential coevolution between bacteria and archaea or bacteriophages in the mammalian gut ecosystem.

However, we found that some fungi genera (e.g., some *Ascomycota* genera) showed the predicted competition mainly with some bacterial genera (e.g., from *Proteobacteria* and *Firmicutes*) in six mammal groups (including GPC, YSNM, RP, CA, OC, and HE; Figures 4 and 5; Appendix S1), but the specific fungi genera were different in each mammalian group (Appendix S1). For example, in GPC, *Beauveria*, *Bipolaris*, *Corynespora*, and *Trematosphaeria* were the primary fungal genera showing a significant negative relationship with some Proteobacteria and Actinobacteria genera (Figure 4b and Appendix S1). In YSNMs, the fungal genus *Phialophora* mainly showed a significant negative relationship with some *Proteobacteria* genera (Figure 4c). In the RP group, some fungal genera showed a significant negative relationship with some genera of bacteria, archaea, and nematodes (Figure 4d and Appendix S1). It should be noted that the sample size from the RP was small (including six samples only), which might lead to biases in co‐occurrence analyses. In CA, *Polyporus* and *Xylona* were the main fungal genera showing a significant negative relationship with some genera from *Firmicutes* (Figure 5a and Appendix S1). In OC, *Ascochyta*, *Monilinia*, and *Tremella* were the main fungal genera showing a significant negative relationship with some genera from *Proteobacteria* and *Firmicutes* (Figure 5b and Appendix S1). The interaction between fungi and bacteria in the gut is complicated, and most of them show a friendly relationship (Sam et al., 2017), as found in our study (significant positive: most co‐occurrence networks). A healthy animal with an intact microbiome can be resistant to pathogenic fungi colonization such as that by *Candida albicans* (Van der Waaij et al., 1971). Interestingly, *Lactobacillus* and *C*. *albicans* may antagonistically compete in the murine digest system (Savage, 1969). Anaerobic gut fungi are important members of the gut microbiome of herbivores, existing in small numbers relative to bacteria, and can degrade cellulose and produce a wealth of secondary metabolites (e.g., they may show antibiotic activity) that may regulate the gut microbiome (Henske et al., 2018; Swift et al., 2019). Therefore, we deduced that the small amount of the predicted competition between fungi and bacteria in the mammalian gut ecosystem might be caused by niche overlap (competition for diet) and antifungal/antibiotic metabolites.

In addition, we found that many protist and nematode genera showed a significantly positive relationship with many other genera, including bacteria, archaea, and fungi, in GPW, GPC, YSNM, CA, OC, and HE (Figures 4 and 5; Appendix S1). Nonhuman primate gut‐associated protists and nematodes are weakly structured by primate phylogeny, with a minimal signal from the diet, which indicates that gut‐associated eukaryotes offer different information than gut‐associated bacteria (Mann et al., 2020). We also found “convergence” at the family level between protists and nematodes in mammal groups, including carnivores, omnivores, and herbivores, regardless of their diet type or the host species.

Gut eukaryotes modulate other microbes through predation, resource and niche competition, and interactions with the host immune system (Lukeš et al., 2015; Stensvold & van der Giezen, 2018). Bacteria are one of the main components of the diets of protists and bacteria (Fu et al., 2005; Saleem et al., 2013). Here, except in the RP group (which may have a potential bias due to the small sample size), complex networks suggest putative mutualistic relationships between mammalian gut eukaryotes (fungi, protists, and nematodes) and prokaryotes (archaea and bacteria). However, parasitic protists (*Plasmodium*) and nematodes (*Onchocercidae* and *Trichuridae*) may differ in their interactions with the host and have different roles in the intestine compared with those of fungi, most of which may play a role in dietary component digestion. In addition, some fungi can prey on nematodes (Dijksterhuis et al., 1994). We found that, in RP, *Onchocerca* spp. had a significantly negative relationship with some fungi genera, including *Rasamsonia*, *Emmonsia*, *Chaetomium*, *Hysterangium*, *Rhodotorula*, *Puccinia*, *Phaffia*, *Naganishia*, and *Kwoniella*. In GPC, *Kytococcus* (*Actinobacteria*) showed a significant negative correlation with *Trichuris*. In OC, *Trichuris* showed a significant negative correlation with some *Proteobacteria* (e.g., *Mesosutterella* and *Rhizobacter*). In HE, the protist parasite *Hepatocystis* showed a significantly negative relationship with some prokaryotes, such as *Methanoculleus*, *Duncaniella*, *Hornefia*, *Calorimonas*, *Breznakia*, and *Gilliamella*. *Hepatocystis* (within the family *Plasmodiidae*) parasites infect monkeys, bats, squirrels, and ungulates in Africa, Asia, and Australia (Ejotre et al., 2021). One study reported coinfection and cross‐species transmission of divergent *Hepatocystis* lineages in a wild African primate community (Thurber et al., 2013). This protist lives with other microorganisms and can predate and kill bacteria in the environment; however, bacteria are also able to resist this action, resulting in profound changes to the protist lifestyle (Henriquez et al., 2021). Thus, we deduced that some prokaryotes might have a potential antiprotist role in the mammalian gut ecosystem.

Here, the reconstruction of mammalian gut microorganism interactions (the predicted mutualism patterns) provides a basic but fresh view of mammalian gut ecosystems, but this does not indicate that parasitic fungi, protists, and nematodes are an important part of the gut ecosystem. In this study, we cannot answer the question of whether these potential parasites are key to maintaining the balance of gut microorganisms.

## 3 CONCLUSION

Metagenomic technology has made considerable progress in gut microbial research. However, most researchers have subconsciously focused on the function of bacteria when using metagenomes. Therefore, we neglected the core advantages of this method: potentially recovering and completing the sequencing of genetic material (including that of organisms from different kingdoms) extracted directly from environmental samples. Implying that we could explore putative interactions among different kingdom organisms in the intestine. Here, we conducted a primary study and revealed some basic features of the mammalian gut ecosystem. We observed high variation in the bacterial and fungal families and relatively low variation (convergence pattern) in those of archaea, viruses, and protist‐nematodes, which reflects the effects of life history. Although we know the limitations of metagenomic analyses in species identification, mainly due to incomplete or short contigs obtained from complex genetic material, we found a potential threat by parasitic protists and nematodes in the studied mammals (in both wild and captive populations). The complex networks among the six microorganism kingdoms studied (archaea, bacteria, viruses, fungi, protists, and nematodes) have deciphered the predicted mutualism in the gut ecosystem. However, we still need to understand the potential competition among gut‐associated eukaryotes, prokaryotes, and viruses.

## 4 MATERIALS AND METHODS

### 4.1 Data used

We used raw datasets of 123 gut metagenomes from 42 mammalian species (including carnivores, omnivores, and herbivores); most of these metagenomes (approximately 104 metagenomes) came from our laboratory using the same sequencing platform to decrease sequencing bias (Appendix S1). From these 123 metagenomes, 52 belonged to giant pandas (9 GPQIN (Wu et al., 2017), 7 GPQIO (Guo et al., 2019), 19 GPXXL (Yao et al., 2021; Zhu, Yang, et al., 2018), 7 GPCD (Zhang et al., 2018), and 10 GPYA) (Guo et al., 2019), 6 to RP, 19 to CA (Zhu, Wu, et al., 2018), 10 to OC (Guo et al., 2018; Zhu, Wu, et al., 2018), 12 to HE (Zhu, Wu, et al., 2018), and 24 to YSNM (Xia et al., 2022).

### 4.2 Metagenomic analyses

The raw reads of the 123 metagenomes were trimmed using Trimmomatic (Bolger et al., 2014) to remove all reads <50 bp in length and reads with degenerate bases (N's). All duplicates were defined as sequences in which the initial 20 nucleotides were identical and shared an overall identity of more than 97% throughout the length of the shortest read. Megahit (Li et al., 2015) was used to assemble the clean reads, and Salmon was used for quality control of the contigs and to remove contigs with coverage below 60% (Patro et al., 2017). We used BWA (Li, 2013) to clean the host genome and delete potential host contaminants. All coding regions (CDS) of metagenomic contigs were predicted by Prodigal (Hyatt et al., 2012) and clustered using CD‐HIT (identity: 95%; overlap: 90%) (Fu et al., 2012), generating unigene pools. Salmon (Patro et al., 2017) was used to map the clean reads to each unigene pool and calculate the transcripts per million (TPM) to determine unigene abundance. Diamond (Buchfink et al., 2015) was used to conduct the alignment of unigenes against the NCBI micro‐NR database (including bacteria, fungi, archaea, viruses, protists, and nematodes) and obtained the TPM of each taxon group using our own software.

### 4.3 Alpha and beta diversity analysis

The TPM of each taxon group was used to calculate Shannon's index. We applied NMDS (nonmetric multidimensional scaling) ordination and the Adonis test in the VEGAN package (Dixon, 2003) based on Bray–Curtis dissimilarity matrices (Beals, 1984) using the TPMs of the taxon groups.

### 4.4 Reconstructing the interactions among microorganism kingdoms

We used our own Perl commands (Appendix S1) to calculate the Spearman correlation (Myers & Sirois, 2004) based on the TPM of the genera of the microorganism kingdoms, and the results were input to Cytoscape (Shannon et al., 2003) for visualization. Here, we used the family level for this calculation to decrease bias in the taxonomic assignment. Overall, we set up the following strict criteria for interaction visualization analysis: (1) all the genera (family in viruses) should be identified in six or more samples (the RP group has six samples only); (2) Spearman correlation *r* ≥ .8 (0.9 for HE, OC, RP, and YSNM due to excessive relationships); (3) *p* ≤ .01; and (4) the interactions among kingdoms were kept, whereas the relationships within each kingdom were excluded.

## AUTHOR CONTRIBUTIONS

**Qinlong Dai:** Formal analysis (equal); visualization (equal); writing – original draft (equal). **Jingjing Ding:** Visualization (equal); writing – original draft (equal). **Xinyuan Cui:** Formal analysis (equal); visualization (equal); writing – original draft (equal). **Yudong Zhu:** Formal analysis (equal); visualization (equal); writing – original draft (equal). **Hua Chen:** Visualization (equal). **Lifeng Zhu:** Conceptualization (lead); data curation (lead); formal analysis (lead); methodology (lead); resources (equal); visualization (lead); writing – original draft (lead).

## CONFLICT OF INTEREST STATEMENT

The authors declared no conflicts of interest relevant to this manuscript.

## Supporting information


Appendix S1

## Data Availability

This is not applicable for this study. The data used in this study come from the published data from several research groups. The personal script used in this study has been included in the Appendix S1.
