# Supplementary material for: Beyond bacteria: Reconstructing microorganism connections and deciphering the predicted mutualisms in mammalian gut metagenomes
Source: Ecol Evol. 2023 Feb 22;13(2):e9829. doi: 10.1002/ece3.9829 (PMC9944162; doi:10.1002/ece3.9829)
Supplement: Supplementary file 1 — Appendix S1 [file ECE3-13-e9829-s001.pdf]

Supplementary materials

Table S1 Information regarding the samples used in this study*

SampleID	Diet	Living style	Species	Location	Group1	Group2
gSRR5415505	Bamboo-eating	Captive	Giant panda	Yaan	GPC	GPYA[1]
SRR5130540	Bamboo-eating	Captive	Giant panda	Yaan	GPC	GPYA[1]
SRR5415504	Bamboo-eating	Captive	Giant panda	Yaan	GPC	GPYA[1]
SRR5150002	Bamboo-eating	Captive	Giant panda	Yaan	GPC	GPYA[1]
SRR5149981	Bamboo-eating	Captive	Giant panda	Yaan	GPC	GPYA[1]
SRR5149936	Bamboo-eating	Captive	Giant panda	Yaan	GPC	GPYA[1]
SRR5149935	Bamboo-eating	Captive	Giant panda	Yaan	GPC	GPYA[1]
SRR5149886	Bamboo-eating	Captive	Giant panda	Yaan	GPC	GPYA[1]
SRR5130583	Bamboo-eating	Captive	Giant panda	Yaan	GPC	GPYA[1]
SRR5130565	Bamboo-eating	Captive	Giant panda	Yaan	GPC	GPYA[1]
ERX2429102	Bamboo-eating	Captive	Giant panda	Chengdu	GPC	GPCD[2]
ERX2429101	Bamboo-eating	Captive	Giant panda	Chengdu	GPC	GPCD[2]
ERX2429100	Bamboo-eating	Captive	Giant panda	Chengdu	GPC	GPCD[2]
ERX2429099	Bamboo-eating	Captive	Giant panda	Chengdu	GPC	GPCD[2]
ERX2429098	Bamboo-eating	Captive	Giant panda	Chengdu	GPC	GPCD[2]
ERX2429087	Bamboo-eating	Captive	Giant panda	Chengdu	GPC	GPCD[2]
ERX2333382	Bamboo-eating	Captive	Giant panda	Chengdu	GPC	GPCD[2]
SAMC011136	Bamboo-eating	Wild	Giant panda	Qinling	GPW	GPQIN[3]
SAMC011114	Bamboo-eating	Wild	Giant panda	Qinling	GPW	GPQIN[3]
SAMC011133	Bamboo-eating	Wild	Giant panda	Qinling	GPW	GPQIN[3]
SAMC011132	Bamboo-eating	Wild	Giant panda	Qinling	GPW	GPQIN[3]
SAMC011131	Bamboo-eating	Wild	Giant panda	Qinling	GPW	GPQIN[3]
SAMC011127	Bamboo-eating	Wild	Giant panda	Qinling	GPW	GPQIN[3]
SAMC011121	Bamboo-eating	Wild	Giant panda	Qinling	GPW	GPQIN[3]
SAMC011118	Bamboo-eating	Wild	Giant panda	Qinling	GPW	GPQIN[3]
SAMC011115	Bamboo-eating	Wild	Giant panda	Qinling	GPW	GPQIN[3]
SRR5149961	Bamboo-eating	Wild	Giant panda	Qionglai	GPW	GPQIO[1]
SRR5149913	Bamboo-eating	Wild	Giant panda	Qionglai	GPW	GPQIO[1]
SRR5149902	Bamboo-eating	Wild	Giant panda	Qionglai	GPW	GPQIO[1]
SRR5149862	Bamboo-eating	Wild	Giant panda	Qionglai	GPW	GPQIO[1]
SRR5130600	Bamboo-eating	Wild	Giant panda	Qionglai	GPW	GPQIO[1]
SRR5130560	Bamboo-eating	Wild	Giant panda	Qionglai	GPW	GPQIO[1]
SRR5130537	Bamboo-eating	Wild	Giant panda	Qionglai	GPW	GPQIO[1]
20140128-zx-F03	Bamboo-eating	Wild	Giant panda	Xiaoxiangling	GPW	GPXXL[4]
20131124-ZX-F03	Bamboo-eating	Wild	Giant panda	Xiaoxiangling	GPW	GPXXL[4]
50	Bamboo-eating	Wild	Giant panda	Xiaoxiangling	GPW	GPXXL[4]
34	Bamboo-eating	Wild	Giant panda	Xiaoxiangling	GPW	GPXXL[4]
31	Bamboo-eating	Wild	Giant panda	Xiaoxiangling	GPW	GPXXL[4]
9	Bamboo-eating	Wild	Giant panda	Xiaoxiangling	GPW	GPXXL[4]
TT2	Bamboo-eating	Wild	Giant panda	Xiaoxiangling	GPW	GPXXL[4]
19	Bamboo-eating	Wild	Giant panda	Xiaoxiangling	GPW	GPXXL[4]
TT1	Bamboo-eating	Wild	Giant panda	Xiaoxiangling	GPW	GPXXL[4]
LZP	Bamboo-eating	Wild	Giant panda	Xiaoxiangling	GPW	GPXXL[4]
LX3	Bamboo-eating	Wild	Giant panda	Xiaoxiangling	GPW	GPXXL[4]
CDXM40	Bamboo-eating	Wild	Giant panda	Xiaoxiangling	GPW	GPXXL[4]
CDXM39	Bamboo-eating	Wild	Giant panda	Xiaoxiangling	GPW	GPXXL[4]
CDXM27	Bamboo-eating	Wild	Giant panda	Xiaoxiangling	GPW	GPXXL[4]
CDXM18	Bamboo-eating	Wild	Giant panda	Xiaoxiangling	GPW	GPXXL[4]
23	Bamboo-eating	Wild	Giant panda	Xiaoxiangling	GPW	GPXXL[4]
1	Bamboo-eating	Wild	Giant panda	Xiaoxiangling	GPW	GPXXL[5]
6	Bamboo-eating	Wild	Giant panda	Xiaoxiangling	GPW	GPXXL[6]
8	Bamboo-eating	Wild	Giant panda	Xiaoxiangling	GPW	GPXXL[6]
C2	Bamboo-eating	Wild	Red panda	Xiaoxiangling	RP	RP[4]
C18	Bamboo-eating	Wild	Red panda	Xiaoxiangling	RP	RP[4]
C14	Bamboo-eating	Wild	Red panda	Xiaoxiangling	RP	RP[4]
B2-4	Bamboo-eating	Wild	Red panda	Xiaoxiangling	RP	RP[4]
B1-2	Bamboo-eating	Wild	Red panda	Xiaoxiangling	RP	RP[4]
A4	Bamboo-eating	Wild	Red panda	Xiaoxiangling	RP	RP[4]
MJ22813045835_PY	HE	Captive	Argali	Beijing Zoo	HE	HE[7]
MJ22813045835_BTCBY	HE	Captive	Hoolock.gibbon	Beijing Zoo	HE	HE[7]
MJ22813045835_BJZML7	HE	Captive	Père David's deer	Beijing Zoo	HE	HE[7]
LN	HE	Captive	Takin	Beijing Zoo	HE	HE[7]

MJ22813045835_MHL	HE	Captive	Sika.deer	Beijing Zoo	HE	HE[7]
MJ22813045835_MGYL	HE	Captive	Onager	Beijing Zoo	HE	HE[7]
MJ22813045835_MAL	HE	Captive	Red.deer	Beijing Zoo	HE	HE[7]
MJ22813045835_HYH	HE	Captive	Francois's Langur	Beijing Zoo	HE	HE[7]
MJ22813045835_HJ	HE	Captive	Hairy.fronted.muntjac	Beijing Zoo	HE	HE[7]
MJ22813045835_EHL	HE	Captive	Goitered.gazelle	Beijing Zoo	HE	HE[7]
MJ22813045835_DJSH	HE	Captive	Black.snub.nosed.monkey	Beijing Zoo	HE	HE[7]
MJ22813045835_BXCBY	HE	Captive	white.cheeked.gibbon	Beijing Zoo	HE	HE[7]
NM	CA	Captive	Caracal	Beijing ZooC	CA	CA[7]
MJ22813045835_CH	CA	Captive	Red.fox	Beijing Zoo	CA	CA[7]
MJ22813045835_CA	CA	Captive	Dhole	Beijing Zoo	CA	CA[7]
MJ22813045835_BHB-NJ	CA	Captive	Spotted.seal	Nanjing Underwater World Park	CA	CA[7]
LB	CA	Captive	Cheetah	Beijing Zoo	CA	CA[7]
HN13	CA	Captive	Indochinese.leopard	Beijing Zoo	CA	CA[7]
HB	CA	Captive	Black.leopard	Beijing Zoo	CA	CA[7]
FZS	CA	Captive	LionBJ	Beijing Zoo	CA	CA[7]
DBH	CA	Captive	Siberian.tiger	Beijing Zoo	CA	CA[7]
BXL	CA	Captive	Maned.wolf	Beijing Zoo	CA	CA[7]
MJLH	CA	Captive	Bengal.tiger	Beijing Zoo	CA	CA[7]
BLG	CA	Captive	Spotted.hyena	Beijing Zoo	CA	CA[7]
MJ22813045835_YH	CA	Captive	Silver fox	Beijing Zoo	CA	CA[7]
MJ22813045835_SH	CA	Captive	Corsac.fox	Beijing Zoo	CA	CA[7]
MJ22813045835_NMHB-NJ	CA	Captive	Spotted.sealNJ2	Nanjing Underwater World Park	CA	CA[7]
MJ22813045835_LH	CA	Captive	Arctic.fox	Beijing Zoo	CA	CA[7]
MJ22813045835_HW2B	CA	Captive	Jaguar	Beijing Zoo	CA	CA[7]
MJ22813045835_HBHL	CA	Captive	Black.backed.jackal	Beijing Zoo	CA	CA[7]
MJ22813045835_GLG	CA	Captive	Striped hyena	Beijing Zoo	CA	CA[7]
ZH	OC	Captive	Hog.badger	Beijing Zoo	OC	OC[7]
MJ22813045835_MLX	OC	Captive	Sun.bear	Beijing Zoo	OC	OC[7]
MJ22813045835_HX	OC	Captive	Raccoon	Beijing Zoo	OC	OC[7]
SRR5130595	OC	Captive	Black bear	Yaan	OC	OC[8]
SRR5130563	OC	Captive	Black bear	Yaan	OC	OC[8]
SRR5130533	OC	Captive	Black bear	Yaan	OC	OC[8]
SRR5130531	OC	Captive	Black bear	Yaan	OC	OC[8]
SRR5130527	OC	Captive	Black bear	Yaan	OC	OC[8]
MJ22813045835_ZX	OC	Captive	Brow.bear	Beijing Zoo	OC	OC[7]

MJ22813045835_MX	OC	Captive	Kinkajou	Beijing Zoo	OC	OC
X-31	HE	Wild	Yunnan snub-nosed monkey	Yunnan	YSNM	YSNM[6]
X-33	HE	Wild	Yunnan snub-nosed monkey	Yunnan	YSNM	YSNM[6]
X-36	HE	Wild	Yunnan snub-nosed monkey	Yunnan	YSNM	YSNM[6]
X-37	HE	Wild	Yunnan snub-nosed monkey	Yunnan	YSNM	YSNM[6]
X-39	HE	Wild	Yunnan snub-nosed monkey	Yunnan	YSNM	YSNM[6]
X-43	HE	Wild	Yunnan snub-nosed monkey	Yunnan	YSNM	YSNM[6]
X-44	HE	Wild	Yunnan snub-nosed monkey	Yunnan	YSNM	YSNM[6]
X-45	HE	Wild	Yunnan snub-nosed monkey	Yunnan	YSNM	YSNM[6]
X-48	HE	Wild	Yunnan snub-nosed monkey	Yunnan	YSNM	YSNM[6]
X-50	HE	Wild	Yunnan snub-nosed monkey	Yunnan	YSNM	YSNM[6]
X-53	HE	Wild	Yunnan snub-nosed monkey	Yunnan	YSNM	YSNM[6]
X-56	HE	Wild	Yunnan snub-nosed monkey	Yunnan	YSNM	YSNM[6]
X-60	HE	Wild	Yunnan snub-nosed monkey	Yunnan	YSNM	YSNM[6]
X-64	HE	Wild	Yunnan snub-nosed monkey	Yunnan	YSNM	YSNM[6]
X-65	HE	Wild	Yunnan snub-nosed monkey	Yunnan	YSNM	YSNM[6]
X-66	HE	Wild	Yunnan snub-nosed monkey	Yunnan	YSNM	YSNM[6]
W-7	HE	Wild	Yunnan snub-nosed monkey	Yunnan	YSNM	YSNM[6]
W-10	HE	Wild	Yunnan snub-nosed monkey	Yunnan	YSNM	YSNM[6]
W-12	HE	Wild	Yunnan snub-nosed monkey	Yunnan	YSNM	YSNM[6]
W-24	HE	Wild	Yunnan snub-nosed monkey	Yunnan	YSNM	YSNM[6]
W-25	HE	Wild	Yunnan snub-nosed monkey	Yunnan	YSNM	YSNM[6]
W-29	HE	Wild	Yunnan snub-nosed	Yunnan	YSNM	YSNM[6]

			monkey			
W-30	HE	Wild	Yunnan snub-nosed monkey	Yunnan	YSNM	YSNM[6]
W-42	HE	Wild	Yunnan snub-nosed monkey	Yunnan	YSNM	YSNM[6]

*This table is reworked on the table S1 in our previous published study.

CA, meat-eating carnivorans. OC, omnivorous carnivorans. HE, herbivores. GPCD, Chengdu giant panda Breeding center. GPYA, giant panda research center in Yaan. GPQIN, the wild giant pandas in Qinling Mountain. GPQIO, the wild giant pandas in Qionglai Mountain. GPXXL, the wild giant pandas in Xiaoxiangling Mountain. RP, the wild red pandas in Xiaoxiangling Mountain. YSNM, the wild Yunnan snub-nosed monkeys in Yunnan. GPC, the combine of GPYA and GPCD. GPW, The combine of GPQIN, GPQIO, and GPXXL.

Group1, the group for co-occurrence analysis. Group2, the group for composition analysis.

Table S2 The comparisons (paiwise Kruskal-Wallis test) on the relative abundance of the putative *Neocallimastigomycetes* among the mammal groups in this study

	Group	Group	p-value
Neocallimastigomycetes	RP	YSNM	1.72E-06
Neocallimastigomycetes	OC	YSNM	2.11E-06
Neocallimastigomycetes	GPYA	YSNM	2.17E-06
Neocallimastigomycetes	GPQIO	YSNM	2.22E-06
Neocallimastigomycetes	GPCD	YSNM	2.71E-06
Neocallimastigomycetes	CA	YSNM	0.0002812
Neocallimastigomycetes	RP	HE	0.0055308
Neocallimastigomycetes	OC	HE	0.0064634
Neocallimastigomycetes	GPYA	HE	0.006649
Neocallimastigomycetes	GPQIO	HE	0.0067848
Neocallimastigomycetes	GPCD	HE	0.0079493
Neocallimastigomycetes	HE	YSNM	0.0417325
Neocallimastigomycetes	RP	CA	0.0441685
Neocallimastigomycetes	GPQIN	YSNM	0.0463395
Neocallimastigomycetes	CA	OC	0.0564552
Neocallimastigomycetes	GPYA	CA	0.0596813
Neocallimastigomycetes	GPQIO	CA	0.0618907
Neocallimastigomycetes	GPCD	CA	0.0794924
Neocallimastigomycetes	CA	HE	0.1333017
Neocallimastigomycetes	GPXXL	RP	0.1409287
Neocallimastigomycetes	GPXXL	OC	0.1484266
Neocallimastigomycetes	GPXXL	GPYA	0.1498189
Neocallimastigomycetes	GPQIO	GPXXL	0.1508051
Neocallimastigomycetes	GPXXL	GPCD	0.1587225
Neocallimastigomycetes	GPCD	RP	0.2164183
Neocallimastigomycetes	GPQIN	RP	0.2385839
Neocallimastigomycetes	GPQIN	OC	0.2566254
Neocallimastigomycetes	GPQIN	GPYA	0.2600506
Neocallimastigomycetes	GPQIN	GPQIO	0.262474
Neocallimastigomycetes	GPQIN	GPCD	0.282006
Neocallimastigomycetes	RP	OC	0.3236203
Neocallimastigomycetes	GPXXL	CA	0.3358147
Neocallimastigomycetes	GPYA	RP	0.3758138
Neocallimastigomycetes	GPQIO	RP	0.3800004
Neocallimastigomycetes	GPCD	OC	0.4780964
Neocallimastigomycetes	GPQIN	GPXXL	0.4963765
Neocallimastigomycetes	GPXXL	YSNM	0.524825
Neocallimastigomycetes	GPQIN	HE	0.5262328
Neocallimastigomycetes	GPYA	GPCD	0.5728652
Neocallimastigomycetes	GPQIO	GPCD	0.6301518
Neocallimastigomycetes	GPXXL	HE	0.7377902

Neocallimastigomycetes	GPQIN	CA	0.738376
Neocallimastigomycetes	GPQIO	OC	0.8426795
Neocallimastigomycetes	GPYA	OC	0.8988386
Neocallimastigomycetes	GPQIO	GPYA	0.9421881

Table S3 The comparisons (paiwise Kruskal-Wallis test) on the relative abundance of the putative *Methanocorpusculaceae* among the mammal groups in this study

	Group	Group	p-value
Methanocorpusculaceae	GPQIN	YSNM	0.0005463
Methanocorpusculaceae	GPQIN	HE	0.0526828
Methanocorpusculaceae	GPYA	HE	0.056621
Methanocorpusculaceae	HE	YSNM	0.0603502
Methanocorpusculaceae	GPQIN	CA	0.0610389
Methanocorpusculaceae	RP	HE	0.0643886
Methanocorpusculaceae	GPYA	CA	0.0763735
Methanocorpusculaceae	GPQIO	HE	0.0806907
Methanocorpusculaceae	CA	YSNM	0.0905535
Methanocorpusculaceae	GPQIN	OC	0.0909531
Methanocorpusculaceae	OC	HE	0.0928125
Methanocorpusculaceae	RP	CA	0.1118372
Methanocorpusculaceae	GPQIN	GPXXL	0.1159977
Methanocorpusculaceae	GPXXL	HE	0.1176414
Methanocorpusculaceae	GPQIN	GPQIO	0.1387293
Methanocorpusculaceae	GPYA	OC	0.141538
Methanocorpusculaceae	GPXXL	GPYA	0.1562179
Methanocorpusculaceae	GPQIN	RP	0.1850422
Methanocorpusculaceae	OC	YSNM	0.1898171
Methanocorpusculaceae	GPXXL	YSNM	0.1931029
Methanocorpusculaceae	CA	HE	0.2038667
Methanocorpusculaceae	GPQIO	CA	0.2130785
Methanocorpusculaceae	GPQIO	GPYA	0.2188366
Methanocorpusculaceae	GPXXL	RP	0.2522827
Methanocorpusculaceae	RP	OC	0.2877591
Methanocorpusculaceae	GPQIO	YSNM	0.2984879
Methanocorpusculaceae	CA	OC	0.3000938
Methanocorpusculaceae	GPQIN	GPCD	0.3601023
Methanocorpusculaceae	GPYA	GPCD	0.373325
Methanocorpusculaceae	GPCD	YSNM	0.3857971
Methanocorpusculaceae	GPCD	RP	0.3980079
Methanocorpusculaceae	GPQIN	GPYA	0.4140558
Methanocorpusculaceae	GPYA	RP	0.4242344
Methanocorpusculaceae	GPQIO	GPCD	0.4436252
Methanocorpusculaceae	GPQIO	RP	0.4495797
Methanocorpusculaceae	GPYA	YSNM	0.460849
Methanocorpusculaceae	GPCD	OC	0.4741602
Methanocorpusculaceae	GPXXL	CA	0.4964402
Methanocorpusculaceae	GPQIO	GPXXL	0.5125431
Methanocorpusculaceae	GPXXL	GPCD	0.5255729
Methanocorpusculaceae	GPCD	HE	0.6613448

Methanocorpusculaceae	RP	YSNM	0.6631362
Methanocorpusculaceae	GPCD	CA	0.6708379
Methanocorpusculaceae	GPXXL	OC	0.7040665
Methanocorpusculaceae	GPQIO	OC	0.7230925

Table S4 The proportion of the correlation-type (positive or negative) in the co-occurrence analysis in this study

Group	Positive	Negative
GPW	0.99991	0.00009
GPC	0.950134	0.049866
RP	0.88624	0.11376
CA	0.95734	0.04266
OC	0.90373	0.09627
HE	0.87626	0.12374
YSNM	0.99342	0.00658
GPC-GPCD	0.65383	0.34617
GPC-GPYA	0.76492	0.23508
GPW-GPQIN	0.83071	0.16929
GPW-GPQIO	0.69888	0.30112
GPW-GPXXL	0.99959	0.00041

The 123 metagenomes used in these analyses belonged to 52 giant pandas (9 from the Qinling Mountains [GPQIN, wild][3], 7 from the Qionglai Mountains [GPQIO, wild][1], 19 from the Xiaoxiangling Mountains [GPXXL, wild][4,5], 7 from the Chengdu Breeding Center [GPCD, captive][2], and 10 from the Yaan research base of the Wolong Research Center [GPYA, captive])[1]; 6 red pandas from the Xiaoxiangling Mountains (RP, wild) [12]; 19 meat-eating carnivorans (CA)[7]; 10 omnivorous carnivorans (OC)[7,8]; 12 herbivores (HE)[7]; and 24 Yunnan snub-nosed monkeys (YSNM, wild)[6].

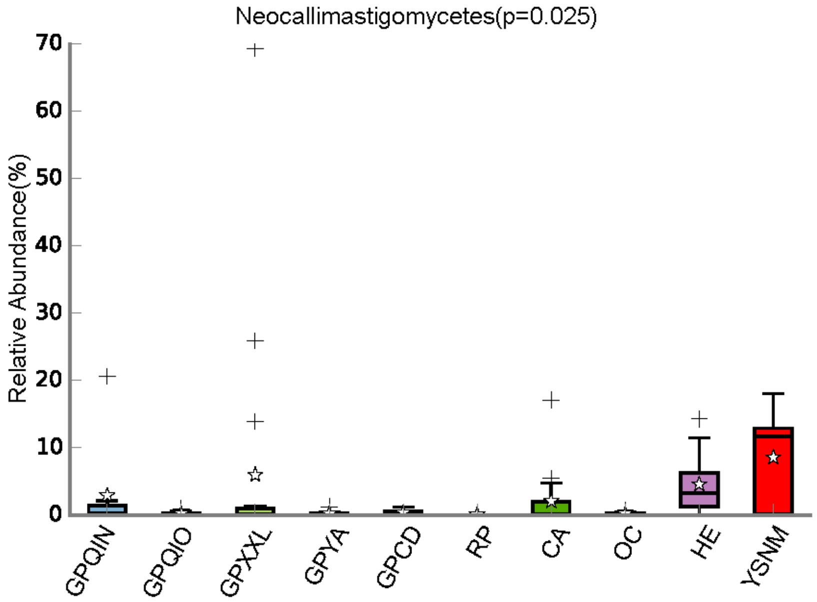

Figure S1 The relative abundance of fungi Neocallimastigomycetes among ten mammal groups

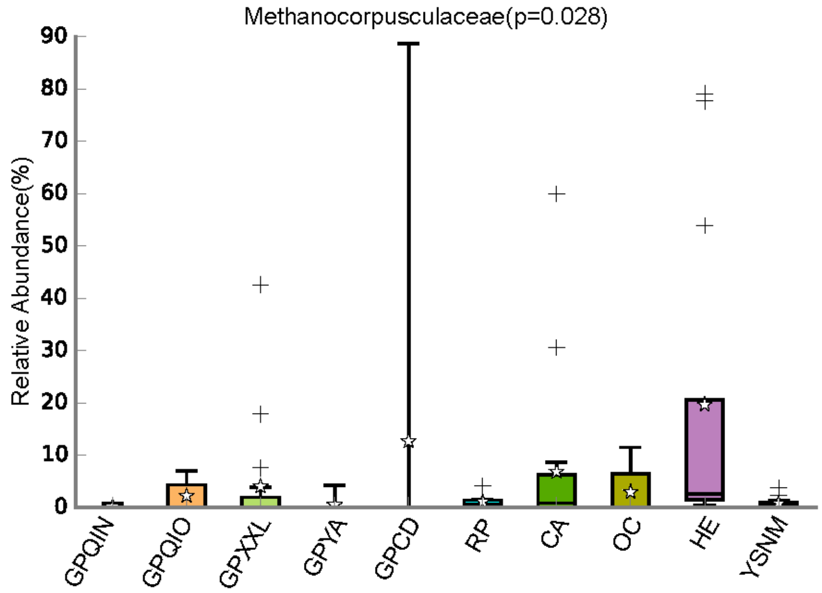

Figure S2 The relative abundance of fungi Methanocorpusculaceae among ten mammal groups

The TPM of total Archaea of GPCD group was lowest among these ten mammal groups. The ‘outlier’ pattern in GPCD caused by the calculation method of relative abundance.

References:

1 Guo, W. *et al.* Comparative study of gut microbiota in wild and captive giant pandas (Ailuropoda melanoleuca). *Genes* **10**, 827 (2019).

2 Zhang, W. *et al.* Age-associated microbiome shows the giant panda lives on hemicelluloses, not on cellulose. *The ISME journal* **12**, 1319-1328 (2018).

3 Wu, Q. *et al.* Seasonal variation in nutrient utilization shapes gut microbiome structure and function in wild giant pandas. *Proceedings of the Royal Society B: Biological Sciences* **284**, 20170955 (2017).

4 Zhu, L. *et al.* Potential mechanism of detoxification of cyanide compounds by gut microbiomes of bamboo-eating pandas. *MSphere* **3**, e00229-00218 (2018).

5 Yao, R. *et al.* Fly-over phylogeny across invertebrate to vertebrate: The giant panda and insects share a highly similar gut microbiota. *Computational and structural biotechnology journal* **19**, 4676-4683 (2021).

6 Xia, W. *et al.* Functional convergence of Yunnan snub-nosed monkey and bamboo-eating panda gut microbiomes revealing the driving by dietary flexibility on mammal gut microbiome. *Computational and Structural Biotechnology Journal* (2022).

7 Zhu, L. *et al.* Adaptive evolution to a high purine and fat diet of carnivorans revealed by gut microbiomes and host genomes. *Environmental microbiology* **20**, 1711-1722 (2018).

8 Guo, W. *et al.* Metagenomic study suggests that the gut microbiota of the giant panda (Ailuropoda melanoleuca) may not be specialized for fiber fermentation. *Frontiers in microbiology* **9**, 229 (2018).

Supplementary excel file 1

The co-occurrence network for each mammal group

(1) GPC (captive giant panda group)

source	target	weight	cor-type	p-vaule	connection	source-type	source-genus(family)	source-degree	target-type	target-abundance	target-genus(family)	target-degree
virus6	bac2882	-0.8222696	negative	5.10E-05	External	virus	f__Genomoviridae	12	bac	5.892754093	g__Arsenophonus	10
virus15	bac2882	-0.8222696	negative	5.10E-05	External	virus	f__Poxviridae	14	bac	5.892754093	g__Arsenophonus	10
virus23	bac2882	-0.825015	negative	4.58E-05	External	virus	f__Ackermannviridae	8	bac	5.892754093	g__Arsenophonus	10
virus26	bac2861	-0.8689656	negative	5.99E-06	External	virus	f__Demerecviridae	43	bac	5.820367756	g__Shimwellia	15
virus34	bac2882	-0.8480434	negative	1.71E-05	External	virus	f__Schitoviridae	6	bac	5.892754093	g__Arsenophonus	10
virus35	bac2880	-0.8406863	negative	5.49E-06	External	virus	f__Siphoviridae	4	bac	5.680298881	g__Obesumbacterium	1
arc80	bac1261	-0.8469926	negative	1.79E-05	External	arc	g__Methanococcoides	37	bac	9.454382577	g__Lactiplantibacillus	1
arc80	bac2861	-0.8266068	negative	4.30E-05	External	arc	g__Methanococcoides	37	bac	5.820367756	g__Shimwellia	15
bac14	fungi291	-0.8117166	negative	7.61E-05	External	bac	g__Terriglobus	5	fungi	6.915708568	g__Beauveria	35
bac84	fungi291	-0.8651961	negative	0	External	bac	g__Rhodococcus	1	fungi	6.915708568	g__Beauveria	35
bac94	fungi291	-0.8073911	negative	8.91E-05	External	bac	g__Blastococcus	2	fungi	6.915708568	g__Beauveria	35
bac121	fungi291	-0.8362927	negative	2.88E-05	External	bac	g__Oerskovia	1	fungi	6.915708568	g__Beauveria	35
bac162	fungi291	-0.8341958	negative	3.14E-05	External	bac	g__Terrabacter	13	fungi	6.915708568	g__Beauveria	35
bac182	fungi291	-0.8004427	negative	0.000113704	External	bac	g__Conyzicola	13	fungi	6.915708568	g__Beauveria	35
bac191	fungi291	-0.8014949	negative	0.000109643	External	bac	g__Glaciibacter	3	fungi	6.915708568	g__Beauveria	35
bac192	fungi291	-0.8634895	negative	8.00E-06	External	bac	g__Glaciihabitans	20	fungi	6.915708568	g__Beauveria	35
bac202	fungi291	-0.8137255	negative	9.09E-05	External	bac	g__Leifsonia	10	fungi	6.915708568	g__Beauveria	35
bac214	fungi291	-0.8101264	negative	8.07E-05	External	bac	g__Plantibacter	13	fungi	6.915708568	g__Beauveria	35
bac265	fungi291	-0.8137255	negative	9.09E-05	External	bac	g__Sanguibacter	4	fungi	6.915708568	g__Beauveria	35
bac316	fungi291	-0.8345629	negative	3.10E-05	External	bac	g__Arachnia	8	fungi	6.915708568	g__Beauveria	35
bac334	fungi291	-0.8355169	negative	2.97E-05	External	bac	g__Tessaracoccus	14	fungi	6.915708568	g__Beauveria	35
bac449	fungi291	-0.8273893	negative	4.16E-05	External	bac	g__Patulibacter	18	fungi	6.915708568	g__Beauveria	35
bac2377	fungi291	-0.8809611	negative	3.02E-06	External	bac	g__Hydrogenophaga	3	fungi	6.915708568	g__Beauveria	35
bac2410	fungi291	-0.8161765	negative	7.99E-05	External	bac	g__Massilia	7	fungi	6.915708568	g__Beauveria	35
bac2520	fungi291	-0.8015505	negative	0.000109432	External	bac	g__Dechloromonas	8	fungi	6.915708568	g__Beauveria	35
bac2645	fungi58	-0.8071749	negative	8.98E-05	External	bac	g__Syntrophus	2	fungi	3.816260723	g__Didymella	54
bac2645	fungi277	-0.8188586	negative	5.82E-05	External	bac	g__Syntrophus	2	fungi	4.770481214	g__Colletotrichum	1
bac2838	fungi253	-0.8177753	negative	6.07E-05	External	bac	g__Cedecea	3	fungi	3.441283105	g__Saccharomyces	9
bac2839	fungi86	-0.8330116	negative	3.31E-05	External	bac	g__Citrobacter	2	fungi	3.283876739	g__Bipolaris	33
bac2846	fungi52	-0.8761878	negative	4.00E-06	External	bac	g__Klebsiella	7	fungi	2.795694711	g__Corynespora	57
bac2846	fungi86	-0.8355169	negative	2.97E-05	External	bac	g__Klebsiella	7	fungi	3.283876739	g__Bipolaris	33
bac2846	fungi96	-0.8253943	negative	4.51E-05	External	bac	g__Klebsiella	7	fungi	2.517454508	g__Trematosphaeria	58
bac2846	fungi111	-0.8104639	negative	7.97E-05	External	bac	g__Klebsiella	7	fungi	4.198193937	g__Cyphellophora	55
bac2846	fungi117	-0.8192325	negative	5.74E-05	External	bac	g__Klebsiella	7	fungi	4.394387424	g__Rhinocladiella	70
bac2846	fungi304	-0.80014	negative	0.000114895	External	bac	g__Klebsiella	7	fungi	3.361763312	g__Neonectria	13
bac2847	fungi52	-0.8761878	negative	4.00E-06	External	bac	g__Kluyvera	12	fungi	2.795694711	g__Corynespora	57
bac2847	fungi86	-0.8705911	negative	5.48E-06	External	bac	g__Kluyvera	12	fungi	3.283876739	g__Bipolaris	33
bac2847	fungi96	-0.8584101	negative	1.04E-05	External	bac	g__Kluyvera	12	fungi	2.517454508	g__Trematosphaeria	58
bac2847	fungi111	-0.8092113	negative	8.34E-05	External	bac	g__Kluyvera	12	fungi	4.198193937	g__Cyphellophora	55
bac2847	fungi116	-0.8229179	negative	4.98E-05	External	bac	g__Kluyvera	12	fungi	4.556746455	g__Phialophora	68
bac2847	fungi117	-0.8129692	negative	7.27E-05	External	bac	g__Kluyvera	12	fungi	4.394387424	g__Rhinocladiella	70
bac2847	fungi335	-0.8025373	negative	0.000105741	External	bac	g__Kluyvera	12	fungi	3.741471904	g__Podospora	11
bac2847	fungi336	-0.8040321	negative	0.000100349	External	bac	g__Kluyvera	12	fungi	3.961289247	g__Thermothelomyces	22
bac2847	fungi480	-0.8104639	negative	7.97E-05	External	bac	g__Kluyvera	12	fungi	5.736265799	g__Lentinus	39
bac2847	fungi602	-0.8177423	negative	6.08E-05	External	bac	g__Kluyvera	12	fungi	7.526776795	g__Phycomyces	9
bac2848	fungi52	-0.8152356	negative	6.68E-05	External	bac	g__Kosakonia	4	fungi	2.795694711	g__Corynespora	57
bac2848	fungi86	-0.8129692	negative	7.27E-05	External	bac	g__Kosakonia	4	fungi	3.283876739	g__Bipolaris	33
bac2848	fungi96	-0.812696	negative	7.34E-05	External	bac	g__Kosakonia	4	fungi	2.517454508	g__Trematosphaeria	58
bac2854	fungi52	-0.8660291	negative	7.01E-06	External	bac	g__Pluralibacter	3	fungi	2.795694711	g__Corynespora	57
bac2854	fungi86	-0.8179798	negative	6.02E-05	External	bac	g__Pluralibacter	3	fungi	3.283876739	g__Bipolaris	33
bac2855	fungi58	-0.8015949	negative	0.000109264	External	bac	g__Pseudescherichia	2	fungi	3.816260723	g__Didymella	54
bac2857	fungi52	-0.8304737	negative	3.67E-05	External	bac	g__Raoultella	6	fungi	2.795694711	g__Corynespora	57
bac2857	fungi86	-0.8568119	negative	1.12E-05	External	bac	g__Raoultella	6	fungi	3.283876739	g__Bipolaris	33
bac2857	fungi96	-0.8050769	negative	9.67E-05	External	bac	g__Raoultella	6	fungi	2.517454508	g__Trematosphaeria	58
bac2857	fungi113	-0.8015505	negative	0.000109432	External	bac	g__Raoultella	6	fungi	5.03348556	g__Cladophialophora	71
bac2857	fungi480	-0.8380222	negative	2.67E-05	External	bac	g__Raoultella	6	fungi	5.736265799	g__Lentinus	39
bac2861	fungi14	-0.8148171	negative	6.79E-05	External	bac	g__Shimwellia	15	fungi	1.73094733	g__Polychaeton	12
bac2861	fungi26	-0.8734856	negative	4.66E-06	External	bac	g__Shimwellia	15	fungi	4.093577602	g__Cercospora	45
bac2861	fungi52	-0.8333026	negative	3.27E-05	External	bac	g__Shimwellia	15	fungi	2.795694711	g__Corynespora	57
bac2861	fungi108	-0.8222696	negative	5.10E-05	External	bac	g__Shimwellia	15	fungi	2.646387541	g__Cryomyces	29
bac2861	fungi111	-0.8079385	negative	8.73E-05	External	bac	g__Shimwellia	15	fungi	4.198193937	g__Cyphellophora	55
bac2861	fungi112	-0.8540702	negative	1.28E-05	External	bac	g__Shimwellia	15	fungi	4.618708133	g__Capronia	76
bac2861	fungi117	-0.8335467	negative	3.23E-05	External	bac	g__Shimwellia	15	fungi	4.394387424	g__Rhinocladiella	70
bac2861	fungi335	-0.8216207	negative	5.23E-05	External	bac	g__Shimwellia	15	fungi	3.741471904	g__Podospora	11
bac2861	fungi339	-0.8129727	negative	7.27E-05	External	bac	g__Shimwellia	15	fungi	3.520608721	g__Neurospora	36
bac2861	fungi343	-0.8099389	negative	8.12E-05	External	bac	g__Shimwellia	15	fungi	2.30587966	g__Phaeoacremonium	42
bac2861	fungi348	-0.8066581	negative	9.14E-05	External	bac	g__Shimwellia	15	fungi	3.445565083	g__Hypoxylon	42
bac2861	fungi404	-0.8266068	negative	4.30E-05	External	bac	g__Shimwellia	15	fungi	3.459812142	g__Tulosesus	44
bac2861	fungi480	-0.8540333	negative	1.29E-05	External	bac	g__Shimwellia	15	fungi	5.736265799	g__Lentinus	39
bac2864	fungi86	-0.8229904	negative	4.96E-05	External	bac	g__Trabulsiella	3	fungi	3.283876739	g__Bipolaris	33
bac2864	fungi96	-0.8152356	negative	6.68E-05	External	bac	g__Trabulsiella	3	fungi	2.517454508	g__Trematosphaeria	58
bac2877	fungi336	-0.8288479	negative	3.93E-05	External	bac	g__Edwardsiella	1	fungi	3.961289247	g__Thermothelomyces	22
bac2882	fungi62	-0.8011561	negative	0.000110937	External	bac	g__Arsenophonus	10	fungi	3.252465328	g__Bimuria	31
bac2882	fungi78	-0.8099389	negative	8.12E-05	External	bac	g__Arsenophonus	10	fungi	3.150896725	g__Stagonospora	50
bac2882	fungi112	-0.8138329	negative	7.04E-05	External	bac	g__Arsenophonus	10	fungi	4.618708133	g__Capronia	76
bac2882	fungi113	-0.805363	negative	9.57E-05	External	bac	g__Arsenophonus	10	fungi	5.03348556	g__Cladophialophora	71
bac2882	fungi198	-0.804747	negative	9.79E-05	External	bac	g__Arsenophonus	10	fungi	3.920506915	g__Helotiales_norank	53
bac2882	fungi340	-0.8015581	negative	0.000109403	External	bac	g__Arsenophonus	10	fungi	2.924236272	g__Sordaria	32
bac2891	fungi358	-0.9105263	negative	3.88E-07	External	bac	g__Affinibrenneria	1	fungi	4.987755537	g__Monosporascus	8
bac3118	fungi291	-0.8319751	negative	3.45E-05	External	bac	g__Rhodanobacter	13	fungi	6.915708568	g__Beauveria	35
bac3121	fungi291	-0.8147393	negative	6.81E-05	External	bac	g__Xanthomonadaceae_Unclassified	5	fungi	6.915708568	g__Beauveria	35
bac167	pro178	-0.8602644	negative	9.44E-06	External	bac	g__Kytococcus	1	pro	8.585126008	g__Trichuris	11

(2) YSNM (Yunnan snub-nosed monkey group)

source	target	weight	cor-type	p-vaule	connection	source-type	source-abundance	source-genus(family)	source-degree	target-type	target-abundance	target-genus(family)	target-degree
arc13	bac614	-0.9043478	negative	2.67E-06	External	arc	6.136077747	g__Candidatus Woesearchaeota_norank	16	bac	4.590371219	g__Salegentibacter	1
arc13	bac1519	-0.9074205	negative	9.57E-10	External	arc	6.136077747	g__Candidatus Woesearchaeota_norank	16	bac	2.921547375	g__Lutibaculum	3
arc38	bac281	-0.9042739	negative	1.36E-09	External	arc	2.425245106	g__Halobiforma	2	bac	3.563627974	g__Sciscionella	2
arc38	bac652	-0.935787	negative	1.96E-11	External	arc	2.425245106	g__Halobiforma	2	bac	5.764325525	g__Pseudosphingobacterium	2
arc78	bac1504	-0.9052174	negative	2.66E-06	External	arc	6.124195307	g__Thermoplasmatales_norank	2	bac	7.980790923	g__Agrobacterium	1
arc79	bac1494	-0.9243478	negative	2.42E-06	External	arc	6.624228561	g__Thermoplasmata_norank	5	bac	8.204493574	g__Mesorhizobium	1
bac390	fungi207	-0.902509	negative	1.65E-09	External	bac	8.452013398	g__Odoribacter	1	fungi	4.343506676	g__Sporothrix	9
bac999	fungi103	-0.9041096	negative	1.39E-09	External	bac	11.43611579	g__Clostridiales_Unclassified	1	fungi	5.633331398	g__Phialophora	14
bac1061	fungi103	-0.9032399	negative	1.52E-09	External	bac	7.083016134	g__Aminicella	1	fungi	5.633331398	g__Phialophora	14
bac1062	fungi24	-0.9073651	negative	9.63E-10	External	bac	7.293641295	g__Anaerofustis	1	fungi	4.827769589	g__Paraphaeosphaeria	2
bac1081	fungi103	-0.9271581	negative	7.52E-11	External	bac	10.46189813	g__Lachnospiraceae_Unclassified	1	fungi	5.633331398	g__Phialophora	14
bac1084	fungi91	-0.9002851	negative	2.09E-09	External	bac	7.819452678	g__Agathobacter	1	fungi	4.805086051	g__Botrytis	11
bac1091	fungi103	-0.909763	negative	7.30E-10	External	bac	8.870000144	g__Bariatricus	1	fungi	5.633331398	g__Phialophora	14
bac1094	fungi103	-0.913242	negative	4.81E-10	External	bac	7.168888786	g__Catonella	1	fungi	5.633331398	g__Phialophora	14
bac1110	fungi103	-0.9006306	negative	2.02E-09	External	bac	11.7573585	g__Lachnoclostridium	1	fungi	5.633331398	g__Phialophora	14
bac1127	fungi103	-0.9171559	negative	2.95E-10	External	bac	9.192545885	g__Tyzzerella	2	fungi	5.633331398	g__Phialophora	14
bac1128	fungi78	-0.9060899	negative	1.11E-09	External	bac	12.30368666	g__Oscillibacter	1	fungi	3.903083164	g__Erysiphe	8
bac1149	fungi24	-0.9117464	negative	5.77E-10	External	bac	5.772711344	g__Acetoanaerobium	1	fungi	4.827769589	g__Paraphaeosphaeria	2
bac1153	fungi30	-0.9191304	negative	2.50E-06	External	bac	5.437352958	g__Filifactor	1	fungi	6.769852058	g__Alternaria	1
bac1176	fungi103	-0.9115025	negative	5.94E-10	External	bac	8.004349199	g__Candidatus Soleaferrea	1	fungi	5.633331398	g__Phialophora	14
bac1187	fungi103	-0.9006306	negative	2.02E-09	External	bac	9.078959808	g__Massilimaliae	1	fungi	5.633331398	g__Phialophora	14
bac1213	fungi308	-0.9045432	negative	1.32E-09	External	bac	4.135470511	g__Levyella	1	fungi	4.037350841	g__Trichosporon	2
bac1260	fungi103	-0.9288976	negative	5.81E-11	External	bac	6.832109972	g__Candidatus Stoquefichus	1	fungi	5.633331398	g__Phialophora	14
bac1266	fungi103	-0.9088933	negative	8.08E-10	External	bac	9.057315641	g__Erysipelatoclostridium	1	fungi	5.633331398	g__Phialophora	14
bac1280	fungi98	-0.9025457	negative	1.64E-09	External	bac	5.882806935	g__Merdibacter	1	fungi	5.506408192	g__Rhynchosporium	3
bac1291	fungi31	-0.9104348	negative	2.61E-06	External	bac	7.007845833	g__Anaerovibrio	1	fungi	5.305972987	g__Bipolaris	1
bac1317	fungi103	-0.9202001	negative	1.98E-10	External	bac	6.956804457	g__Dialister	1	fungi	5.633331398	g__Phialophora	14
bac1333	fungi93	-0.9460404	negative	3.03E-12	External	bac	6.39720795	g__Murdochiella	1	fungi	5.741071837	g__Sclerotinia	9
bac1978	fungi3	-0.9006803	negative	2.01E-09	External	bac	5.434376811	g__Bradymonadales_Unclassified	1	fungi	4.625423692	g__Neofusicoccum	3
bac2060	fungi180	-0.9060056	negative	1.12E-09	External	bac	5.143568174	g__Syntrophus	1	fungi	3.883520645	g__Pochonia	1
bac2494	fungi103	-0.9123723	negative	5.35E-10	External	bac	8.097361476	g__Anaeroplasma	1	fungi	5.633331398	g__Phialophora	14

(3) CA (meat-eating carnivorans)

source	target	weight	cor-type	p-vaule	connection	source-type	source-abundance	source-genus(family)	source-degree	target-type	target-abundance	target-genus(family)	target-degree
bac485	fungi481	-0.8309129	negative	1.05E-05	External	bac	8.328129759	g__Duncaniella	1	fungi	6.007505205	g__Polyporus	9
bac499	fungi362	-0.820404	negative	1.69E-05	External	bac	7.679213977	g__Porphyromonas	2	fungi	3.751243738	g__Xylona	7
bac499	fungi481	-0.810511	negative	2.58E-05	External	bac	7.679213977	g__Porphyromonas	2	fungi	6.007505205	g__Polyporus	9
bac514	fungi267	-0.8093585	negative	2.70E-05	External	bac	6.683472329	g__Millionella	1	fungi	4.733941517	g__Coniochaeta	7
bac1110	fungi481	-0.8354922	negative	8.48E-06	External	bac	3.656717767	g__Peribacillus	1	fungi	6.007505205	g__Polyporus	9
bac1298	fungi121	-0.8018013	negative	3.66E-05	External	bac	6.026095416	g__Candidatus Arthromitus	1	fungi	4.497328818	g__Aspergillus	5
bac1353	fungi362	-0.804428	negative	3.30E-05	External	bac	4.509616977	g__Hornefia	1	fungi	3.751243738	g__Xylona	7
bac1367	fungi267	-0.8255587	negative	1.35E-05	External	bac	7.281859743	g__Anaerocolumna	2	fungi	4.733941517	g__Coniochaeta	7
bac1367	fungi448	-0.8087635	negative	2.77E-05	External	bac	7.281859743	g__Anaerocolumna	2	fungi	3.691825111	g__Hydnum	3
bac1416	fungi481	-0.8142204	negative	2.21E-05	External	bac	6.629512516	g__Pseudobutyrivibrio	1	fungi	6.007505205	g__Polyporus	9
bac1417	fungi481	-0.8735714	negative	1.04E-06	External	bac	6.843855487	g__Robinsoniella	2	fungi	6.007505205	g__Polyporus	9
bac1420	fungi601	-0.822807	negative	1.01E-05	External	bac	7.273662177	g__Sellimonas	1	fungi	5.749176402	g__Thamnidium	1
bac1432	fungi339	-0.8172245	negative	1.94E-05	External	bac	6.856425142	g__Acutalibacter	1	fungi	3.313676784	g__Neurospora	8
bac1474	fungi362	-0.8276005	negative	1.23E-05	External	bac	8.746856721	g__Subdoligranulum	3	fungi	3.751243738	g__Xylona	7
bac1474	fungi481	-0.8160752	negative	2.04E-05	External	bac	8.746856721	g__Subdoligranulum	3	fungi	6.007505205	g__Polyporus	9
bac1684	fungi362	-0.8225003	negative	1.54E-05	External	bac	4.104377398	g__Sporanaerobacter	2	fungi	3.751243738	g__Xylona	7
bac1696	fungi177	-0.8006556	negative	3.83E-05	External	bac	11.462515	g__Cetobacterium	1	fungi	2.735211309	g__Cudoniella	4
bac3025	fungi216	-0.8534245	negative	3.39E-06	External	bac	5.80320238	g__Actinobacillus	4	fungi	1.973108331	g__Tirmania	11
bac3025	fungi222	-0.8140351	negative	2.20E-05	External	bac	5.80320238	g__Actinobacillus	4	fungi	5.065256046	g__Wilcoxina	3
bac3025	fungi267	-0.816748	negative	1.98E-05	External	bac	5.80320238	g__Actinobacillus	4	fungi	4.733941517	g__Coniochaeta	7
bac3025	fungi337	-0.8580107	negative	2.63E-06	External	bac	5.80320238	g__Actinobacillus	4	fungi	3.598817976	g__Thermothielavioides	10
bac3099	fungi362	-0.8173432	negative	1.93E-05	External	bac	4.180476386	g__Photobacterium	2	fungi	3.751243738	g__Xylona	7
bac3099	fungi481	-0.8349871	negative	8.69E-06	External	bac	4.180476386	g__Photobacterium	2	fungi	6.007505205	g__Polyporus	9
bac3179	fungi481	-0.8272035	negative	1.25E-05	External	bac	7.522604645	g__Treponema	1	fungi	6.007505205	g__Polyporus	9

(4) OC (Omnivorous carnivorans)

source	target	weight	cor-type	p-vaule	connection	source-type	source-abu	source-genus(family)	source-degree	target-type	target-abundance	target-genu	target-degree
bac1063	fungi535	-0.903226	negative	0.0003409	External	bac	2.2178983	g__Amphibacillus	1	fungi	3.627402907	g__Tremella	8
bac1070	fungi212	-0.922581	negative	0.000143	External	bac	5.2147401	g__Caldalkalibacillus	9	fungi	2.747783603	g__Ascodes	3
bac1070	fungi219	-0.947086	negative	3.22E-05	External	bac	5.2147401	g__Caldalkalibacillus	9	fungi	4.817163807	g__Pyronem	10
bac1070	fungi221	-0.917887	negative	0.00018	External	bac	5.2147401	g__Caldalkalibacillus	9	fungi	4.525639847	g__Trichoph	21
bac1070	fungi267	-0.917887	negative	0.00018	External	bac	5.2147401	g__Caldalkalibacillus	9	fungi	5.902390134	g__Coniocha	13
bac1070	fungi300	-0.905227	negative	0.0003144	External	bac	5.2147401	g__Caldalkalibacillus	9	fungi	7.481625559	g__Fusarium	1
bac1070	fungi316	-0.922581	negative	0.000143	External	bac	5.2147401	g__Caldalkalibacillus	9	fungi	5.429687676	g__Pyricular	4
bac1070	fungi335	-0.943208	negative	4.25E-05	External	bac	5.2147401	g__Caldalkalibacillus	9	fungi	6.092677724	g__Podospo	13
bac1070	fungi342	-0.944211	negative	3.96E-05	External	bac	5.2147401	g__Caldalkalibacillus	9	fungi	4.879044874	g__Madurel	5
bac1070	fungi549	-0.922581	negative	0.000143	External	bac	5.2147401	g__Caldalkalibacillus	9	fungi	5.991442047	g__Ustilago	3
bac1119	fungi535	-0.903226	negative	0.0003409	External	bac	5.0838424	g__Robertmurraya	1	fungi	3.627402907	g__Tremella	8
bac1214	fungi535	-0.906693	negative	0.0002959	External	bac	8.4518947	g__Lactobacillales_Unclassified	1	fungi	3.627402907	g__Tremella	8
bac1262	fungi535	-0.969223	negative	3.78E-06	External	bac	11.601024	g__Lactobacillus	1	fungi	3.627402907	g__Tremella	8
bac1270	fungi535	-0.919199	negative	0.000169	External	bac	7.4074494	g__Liquorilactobacillus	1	fungi	3.627402907	g__Tremella	8
bac1274	fungi535	-0.925452	negative	0.0001234	External	bac	6.5727719	g__Pediococcus	1	fungi	3.627402907	g__Tremella	8
bac1282	fungi535	-0.925452	negative	0.0001234	External	bac	14.114406	g__Streptococcus	1	fungi	3.627402907	g__Tremella	8
bac1290	fungi600	-0.903226	negative	0.0003409	External	bac	4.9492633	g__Alkaliphilus	1	fungi	4.357567432	g__Parasitel	1
bac1292	fungi325	-0.901908	negative	0.0003593	External	bac	5.0468258	g__Anaerophilus	1	fungi	10.99602801	g__Sporothr	13
bac1321	fungi221	-0.938721	negative	5.73E-05	External	bac	12.972085	g__Sarcina	1	fungi	4.525639847	g__Trichoph	21
bac1574	fungi267	-0.910808	negative	0.0002483	External	bac	4.4300665	g__Thermoanaerobacterium	3	fungi	5.902390134	g__Coniocha	13
bac1574	fungi335	-0.923116	negative	0.0001392	External	bac	4.4300665	g__Thermoanaerobacterium	3	fungi	6.092677724	g__Podospo	13
bac1574	fungi344	-0.934541	negative	7.42E-05	External	bac	4.4300665	g__Thermoanaerobacterium	3	fungi	3.830596007	g__Arthriniu	5
bac1588	fungi535	-0.903226	negative	0.0003409	External	bac	2.6703483	g__Sharpea	1	fungi	3.627402907	g__Tremella	8
bac2123	fungi190	-0.902439	negative	0.0003518	External	bac	4.3146574	g__Phaeobacter	1	fungi	10.40492023	g__Monilinia	15
bac2562	fungi190	-0.972349	negative	2.47E-06	External	bac	4.3281379	g__Desulfoluna	2	fungi	10.40492023	g__Monilinia	15
bac2677	fungi190	-0.957317	negative	1.38E-05	External	bac	4.9416924	g__Dongshaea	1	fungi	10.40492023	g__Monilinia	15
bac2680	fungi190	-0.920732	negative	0.0001568	External	bac	5.6850583	g__Tolumonas	2	fungi	10.40492023	g__Monilinia	15
bac2714	fungi190	-0.923116	negative	0.0001392	External	bac	5.381196	g__Ferrimonas	1	fungi	10.40492023	g__Monilinia	15
bac2718	fungi57	-0.949986	negative	2.58E-05	External	bac	4.0449617	g__Pseudidiomarina	3	fungi	4.230593093	g__Ascochy	31
bac2718	fungi220	-0.914651	negative	0.0002092	External	bac	4.0449617	g__Pseudidiomarina	3	fungi	4.785665075	g__Sphaero	22
bac2718	fungi363	-0.919771	negative	0.0001644	External	bac	4.0449617	g__Pseudidiomarina	3	fungi	4.70663412	g__Saitoella	25
bac2723	fungi57	-0.943208	negative	4.25E-05	External	bac	4.0367684	g__Psychrosphaera	4	fungi	4.230593093	g__Ascochy	31
bac2723	fungi220	-0.938721	negative	5.73E-05	External	bac	4.0367684	g__Psychrosphaera	4	fungi	4.785665075	g__Sphaero	22
bac2723	fungi277	-0.905227	negative	0.0003144	External	bac	4.0367684	g__Psychrosphaera	4	fungi	6.531139984	g__Colletotr	25
bac2723	fungi363	-0.925466	negative	0.0001233	External	bac	4.0367684	g__Psychrosphaera	4	fungi	4.70663412	g__Saitoella	25
bac2729	fungi190	-0.921997	negative	0.0001473	External	bac	4.5745873	g__Motilimonas	1	fungi	10.40492023	g__Monilinia	15
bac2824	fungi187	-0.914179	negative	0.0002137	External	bac	6.3456258	g__Sodalis	4	fungi	9.674532271	g__Botryotir	2
bac2824	fungi188	-0.901908	negative	0.0003593	External	bac	6.3456258	g__Sodalis	4	fungi	11.00885824	g__Botrytis	3
bac2824	fungi193	-0.901908	negative	0.0003593	External	bac	6.3456258	g__Sodalis	4	fungi	10.47670555	g__Sclerotiu	3
bac2827	fungi190	-0.935425	negative	7.03E-05	External	bac	5.413595	g__Insectihabitans	1	fungi	10.40492023	g__Monilinia	15
bac2831	fungi190	-0.911858	negative	0.0002371	External	bac	5.5616235	g__Pragia	2	fungi	10.40492023	g__Monilinia	15
bac2845	fungi190	-0.924016	negative	0.000133	External	bac	5.1182812	g__Jejubacter	1	fungi	10.40492023	g__Monilinia	15
bac2846	fungi97	-0.919199	negative	0.000169	External	bac	10.79131	g__Klebsiella	3	fungi	4.50511778	g__Aaospha	2
bac2846	fungi186	-0.919199	negative	0.000169	External	bac	10.79131	g__Klebsiella	3	fungi	8.280110439	g__Rutstroe	3
bac2877	fungi60	-0.912946	negative	0.000226	External	bac	9.0868953	g__Edwardsiella	3	fungi	3.225930879	g__Macrove	11
bac2877	fungi523	-0.92645	negative	0.0001171	External	bac	9.0868953	g__Edwardsiella	3	fungi	5.24209616	g__Puccinia	6
bac2911	fungi57	-0.931705	negative	8.76E-05	External	bac	12.288642	g__Plesiomonas	4	fungi	4.230593093	g__Ascochy	31
bac2911	fungi190	-0.984807	negative	2.29E-07	External	bac	12.288642	g__Plesiomonas	4	fungi	10.40492023	g__Monilinia	15
bac2911	fungi523	-0.901908	negative	0.0003593	External	bac	12.288642	g__Plesiomonas	4	fungi	5.24209616	g__Puccinia	6
bac3042	fungi188	-0.92645	negative	0.0001171	External	bac	4.4793008	g__Mannheimia	2	fungi	11.00885824	g__Botrytis	3
bac3042	fungi193	-0.92645	negative	0.0001171	External	bac	4.4793008	g__Mannheimia	2	fungi	10.47670555	g__Sclerotiu	3
bac3096	fungi190	-0.957317	negative	1.38E-05	External	bac	5.0059309	g__Enterovibrio	1	fungi	10.40492023	g__Monilinia	15
bac3100	fungi57	-0.944211	negative	3.96E-05	External	bac	5.1618904	g__Salinivibrio	1	fungi	4.230593093	g__Ascochy	31
bac3102	fungi190	-0.935425	negative	7.03E-05	External	bac	3.9341758	g__Veronia	2	fungi	10.40492023	g__Monilinia	15
pro178	fungi187	-0.901908	negative	0.0003593	External	pro	7.9575526	g__Trichuris	11	fungi	9.674532271	g__Botryotir	2
pro178	fungi190	-0.948333	negative	2.93E-05	External	pro	7.9575526	g__Trichuris	11	fungi	10.40492023	g__Monilinia	15
bac119	pro155	-0.914179	negative	0.0002137	External	bac	2.0818137	g__Actinotalea	1	pro	7.996863884	g__Onchoce	14
bac2151	pro155	-0.927273	negative	0.0001302	External	bac	4.9815772	g__Acetobacter	2	pro	7.996863884	g__Onchoce	14
bac2422	pro178	-0.936175	negative	6.72E-05	External	bac	3.312439	g__Mesosutterella	1	pro	7.957552603	g__Trichuris	11
bac2438	pro178	-0.938721	negative	5.73E-05	External	bac	6.7680023	g__Rhizobacter	3	pro	7.957552603	g__Trichuris	11

(5) HE (Herbivores)

source	target	weight	cor-type	p-vaule	connection	source-type	source-abu	source-genus(family)	source-degree	target-type	target-abundance	target-genus(family)	target-degr
virus10	bac1498	-0.922666	negative	1.91E-05	External	virus	5.7108276	f__Phycodnaviridae	2	bac	3.046330326	g__Acetoanaerobium	2
arc17	bac1902	-0.906956	negative	4.69E-05	External	arc	9.0557982	g__Methanomassiliicoccales_norank	1	bac	5.488871608	g__Nitratireductor	3
arc31	bac1511	-0.908473	negative	4.33E-05	External	arc	4.8936444	g__Candidatus Methanofastidiosa_norank	1	bac	3.292943796	g__Tepidibacter	1
arc48	bac479	-0.92935	negative	1.23E-05	External	arc	1.4369875	g__Natronobacterium	11	bac	5.899171062	g__Petrimonas	2
arc63	bac1069	-0.900177	negative	6.59E-05	External	arc	5.2388221	g__Methanoculleus	7	bac	10.38882535	g__Bacillus	2
arc63	bac1070	-0.91769	negative	2.59E-05	External	arc	5.2388221	g__Methanoculleus	7	bac	5.514488668	g__Caldalkalibacillus	1
arc63	bac1902	-0.912285	negative	3.52E-05	External	arc	5.2388221	g__Methanoculleus	7	bac	5.488871608	g__Nitratireductor	3
arc89	bac1415	-0.93007	negative	0	External	arc	6.0932605	g__Methanomicrobia_norank	2	bac	5.733483662	g__Porcincola	1
bac483	fungi65	-0.901431	negative	6.20E-05	External	bac	6.8869891	g__Candidatus Amulumruptor	1	fungi	1.54734972	g__Paraphaeosphaeria	1
bac970	fungi221	-0.906392	negative	4.83E-05	External	bac	3.4437778	g__Cyanothece	1	fungi	3.983208139	g__Trichophaea	1
bac1282	fungi114	-0.906956	negative	4.69E-05	External	bac	9.5169889	g__Streptococcus	1	fungi	1.219649284	g__Exophiala	2
bac1379	fungi342	-0.951049	negative	0	External	bac	6.0157034	g__Catenibacillus	1	fungi	4.183850049	g__Madurella	1
bac1446	fungi325	-0.902098	negative	0	External	bac	10.946514	g__Faecalibacterium	1	fungi	3.205425777	g__Sporothrix	3
bac1540	fungi246	-0.922509	negative	1.93E-05	External	bac	3.6914262	g__Levyella	3	fungi	4.238488195	g__Pichia	1
bac1540	fungi572	-0.904807	negative	5.24E-05	External	bac	3.6914262	g__Levyella	3	fungi	3.63819124	g__Nematocida	1
bac2424	fungi230	-0.911176	negative	3.75E-05	External	bac	8.5519967	g__Sutterella	1	fungi	3.069911672	g__Lodderomyces	4
bac2425	fungi277	-0.951049	negative	0	External	bac	5.3618413	g__Turicimonas	1	fungi	4.274218682	g__Colletotrichum	3
bac2922	fungi230	-0.932532	negative	9.82E-06	External	bac	5.7278961	g__Legionella	1	fungi	3.069911672	g__Lodderomyces	4
bac3042	fungi590	-0.908043	negative	4.43E-05	External	bac	4.5604029	g__Mannheimia	1	fungi	1.968090899	g__Mortierella	1
bac3204	fungi395	-0.906956	negative	4.69E-05	External	bac	7.0743437	g__Mycoplasmopsis	1	fungi	2.036011984	g__Flammulina	2
arc63	pro1	-0.9443	negative	3.84E-06	External	arc	5.2388221	g__Methanoculleus	7	pro	4.479935707	g__Hepatocystis	10
bac479	pro3	-0.942648	negative	4.44E-06	External	bac	5.8991711	g__Petrimonas	2	pro	6.641760332	g__Babesia	3
bac485	pro1	-0.942648	negative	4.44E-06	External	bac	7.7658506	g__Duncaniella	1	pro	4.479935707	g__Hepatocystis	10
bac1353	pro1	-0.913643	negative	3.27E-05	External	bac	6.0600503	g__Hornefia	1	pro	4.479935707	g__Hepatocystis	10
bac1564	pro1	-0.902256	negative	5.95E-05	External	bac	4.2957604	g__Calorimonas	3	pro	4.479935707	g__Hepatocystis	10
bac1597	pro1	-0.906392	negative	4.83E-05	External	bac	3.3942735	g__Breznakia	3	pro	4.479935707	g__Hepatocystis	10
bac3021	pro1	-0.918877	negative	2.41E-05	External	bac	3.3183953	g__Gilliamella	3	pro	4.479935707	g__Hepatocystis	10

(6) RP (Red panda group)

source	target	weight	cor-type	p-vaule	connection	source-type	source-abu	source-genus(family)	source-deg	target-type	target-abundance	target-genus(family)	target-degr
virus30	bac1899	-0.942857	negative	0.0166667	External	virus	8.2318864	f__Myoviridae	4	bac	3.82091598	g__Hoeflea	4
virus34	bac428	-0.942857	negative	0.0166667	External	virus	6.4627892	f__Schitoviridae	4	bac	1.792592456	g__Adlercreutzia	3
virus34	bac1390	-0.942857	negative	0.0166667	External	virus	6.4627892	f__Schitoviridae	4	bac	3.662227423	g__Faecalimonas	3
virus35	bac76	-0.942857	negative	0.0166667	External	virus	8.6570158	f__Siphoviridae	5	bac	5.684919006	g__Mycobacterium	2
arc51	bac94	-0.942857	negative	0.0166667	External	arc	5.5535059	g__Methanobrevibacter	10	bac	3.33829308	g__Blastococcus	4
arc51	bac222	-0.942857	negative	0.0166667	External	arc	5.5535059	g__Methanobrevibacter	10	bac	2.831447079	g__Schumannella	1
virus35	fungi199	-0.942857	negative	0.0166667	External	virus	8.6570158	f__Siphoviridae	5	fungi	3.610431308	g__Antarctomyces	2
arc51	fungi202	-0.942857	negative	0.0166667	External	arc	5.5535059	g__Methanobrevibacter	10	fungi	5.481009688	g__Pseudogymnoascus	11
arc51	fungi343	-0.942857	negative	0.0166667	External	arc	5.5535059	g__Methanobrevibacter	10	fungi	3.590384207	g__Phaeoacremonium	5
arc51	fungi505	-0.942857	negative	0.0166667	External	arc	5.5535059	g__Methanobrevibacter	10	fungi	1.707471653	g__Ceraceosorus	5
arc51	fungi515	-0.942857	negative	0.0166667	External	arc	5.5535059	g__Methanobrevibacter	10	fungi	4.592621783	g__Microbotryum	5
arc51	fungi601	-0.942857	negative	0.0166667	External	arc	5.5535059	g__Methanobrevibacter	10	fungi	4.502783023	g__Thamnidium	5
bac588	fungi202	-0.942857	negative	0.0166667	External	bac	3.8528036	g__Taibaiella	6	fungi	5.481009688	g__Pseudogymnoascus	11
bac588	fungi343	-0.942857	negative	0.0166667	External	bac	3.8528036	g__Taibaiella	6	fungi	3.590384207	g__Phaeoacremonium	5
bac588	fungi505	-0.942857	negative	0.0166667	External	bac	3.8528036	g__Taibaiella	6	fungi	1.707471653	g__Ceraceosorus	5
bac588	fungi515	-0.942857	negative	0.0166667	External	bac	3.8528036	g__Taibaiella	6	fungi	4.592621783	g__Microbotryum	5
bac588	fungi601	-0.942857	negative	0.0166667	External	bac	3.8528036	g__Taibaiella	6	fungi	4.502783023	g__Thamnidium	5
bac661	fungi514	-0.942857	negative	0.0166667	External	bac	2.8258925	g__Emticicia	2	fungi	5.297687168	g__Leucosporidium	4
bac739	fungi113	-0.942857	negative	0.0166667	External	bac	3.6848714	g__Lutibacter	2	fungi	2.768580701	g__Cladophialophora	52
bac739	fungi359	-0.942857	negative	0.0166667	External	bac	3.6848714	g__Lutibacter	2	fungi	3.210722678	g__Phialemoniopsis	52
bac807	fungi195	-0.942857	negative	0.0166667	External	bac	4.2145986	g__Empedobacter	2	fungi	1.453719337	g__Chalara	24
bac807	fungi280	-0.942857	negative	0.0166667	External	bac	4.2145986	g__Empedobacter	2	fungi	2.74970385	g__Verticillium	24
bac1418	fungi128	-0.942857	negative	0.0166667	External	bac	4.3317066	g__Roseburia	6	fungi	2.618927506	g__Rasamsonia	33
bac1418	fungi202	-0.942857	negative	0.0166667	External	bac	4.3317066	g__Roseburia	6	fungi	5.481009688	g__Pseudogymnoascus	11
bac1418	fungi330	-0.942857	negative	0.0166667	External	bac	4.3317066	g__Roseburia	6	fungi	4.362996709	g__Chaetomium	33
bac1418	fungi464	-0.942857	negative	0.0166667	External	bac	4.3317066	g__Roseburia	6	fungi	3.881790576	g__Hysterangium	33
bac1418	fungi516	-0.942857	negative	0.0166667	External	bac	4.3317066	g__Roseburia	6	fungi	5.101347038	g__Rhodotorula	33
bac1418	fungi523	-0.942857	negative	0.0166667	External	bac	4.3317066	g__Roseburia	6	fungi	5.735294062	g__Puccinia	33
bac1634	fungi199	-0.942857	negative	0.0166667	External	bac	2.460187	g__Megamonas	2	fungi	3.610431308	g__Antarctomyces	2
bac1786	fungi335	-0.942857	negative	0.0166667	External	bac	5.5822135	g__Proteobacteria_Unclassified	1	fungi	4.879835406	g__Podospora	5
bac1937	fungi600	-0.942857	negative	0.0166667	External	bac	3.8047273	g__Sinorhizobium	1	fungi	1.616016228	g__Parasitella	7
bac2321	fungi57	-0.942857	negative	0.0166667	External	bac	2.5559086	g__Castellaniella	8	fungi	2.400981415	g__Ascochyta	41
bac2321	fungi195	-1	negative	0.0027778	External	bac	2.5559086	g__Castellaniella	8	fungi	1.453719337	g__Chalara	24
bac2321	fungi212	-0.942857	negative	0.0166667	External	bac	2.5559086	g__Castellaniella	8	fungi	5.614594184	g__Ascodesmis	41
bac2321	fungi277	-0.942857	negative	0.0166667	External	bac	2.5559086	g__Castellaniella	8	fungi	4.265661344	g__Colletotrichum	41
bac2321	fungi280	-1	negative	0.0027778	External	bac	2.5559086	g__Castellaniella	8	fungi	2.74970385	g__Verticillium	24
bac2321	fungi305	-0.942857	negative	0.0166667	External	bac	2.5559086	g__Castellaniella	8	fungi	2.434577802	g__Stylonectria	14
bac2321	fungi322	-0.942857	negative	0.0166667	External	bac	2.5559086	g__Castellaniella	8	fungi	2.021150133	g__Grosmannia	18
bac2321	fungi357	-0.942857	negative	0.0166667	External	bac	2.5559086	g__Castellaniella	8	fungi	3.715935126	g__Xylaria	14
bac2329	fungi181	-0.942857	negative	0.0166667	External	bac	3.2356149	g__Paracandidimonas	10	fungi	3.915058394	g__Lachnellula	34
bac2329	fungi188	-0.942857	negative	0.0166667	External	bac	3.2356149	g__Paracandidimonas	10	fungi	3.46213568	g__Botrytis	34
bac2329	fungi305	-0.942857	negative	0.0166667	External	bac	3.2356149	g__Paracandidimonas	10	fungi	2.434577802	g__Stylonectria	14
bac2329	fungi343	-1	negative	0.0027778	External	bac	3.2356149	g__Paracandidimonas	10	fungi	3.590384207	g__Phaeoacremonium	5
bac2329	fungi357	-0.942857	negative	0.0166667	External	bac	3.2356149	g__Paracandidimonas	10	fungi	3.715935126	g__Xylaria	14
bac2329	fungi505	-1	negative	0.0027778	External	bac	3.2356149	g__Paracandidimonas	10	fungi	1.707471653	g__Ceraceosorus	5
bac2329	fungi514	-0.942857	negative	0.0166667	External	bac	3.2356149	g__Paracandidimonas	10	fungi	5.297687168	g__Leucosporidium	4
bac2329	fungi515	-1	negative	0.0027778	External	bac	3.2356149	g__Paracandidimonas	10	fungi	4.592621783	g__Microbotryum	5
bac2329	fungi601	-1	negative	0.0027778	External	bac	3.2356149	g__Paracandidimonas	10	fungi	4.502783023	g__Thamnidium	5
bac2386	fungi600	-0.942857	negative	0.0166667	External	bac	5.0086467	g__Ottowia	1	fungi	1.616016228	g__Parasitella	7
bac2408	fungi195	-0.942857	negative	0.0166667	External	bac	12.970603	g__Janthinobacterium	2	fungi	1.453719337	g__Chalara	24
bac2408	fungi280	-0.942857	negative	0.0166667	External	bac	12.970603	g__Janthinobacterium	2	fungi	2.74970385	g__Verticillium	24
bac2456	fungi335	-0.942857	negative	0.0166667	External	bac	3.282639	g__Chromobacterium	1	fungi	4.879835406	g__Podospora	5
bac2464	fungi134	-1	negative	0.0027778	External	bac	0.7866375	g__Microvirgula	5	fungi	3.214064268	g__Emmonsia	3
bac2464	fungi514	-0.942857	negative	0.0166667	External	bac	0.7866375	g__Microvirgula	5	fungi	5.297687168	g__Leucosporidium	4
bac2464	fungi526	-0.942857	negative	0.0166667	External	bac	0.7866375	g__Microvirgula	5	fungi	9.289104509	g__Phaffia	10
bac2464	fungi538	-0.942857	negative	0.0166667	External	bac	0.7866375	g__Microvirgula	5	fungi	4.446936181	g__Tremellales_norank	10
bac2477	fungi195	-0.942857	negative	0.0166667	External	bac	2.2416667	g__Crenobacter	4	fungi	1.453719337	g__Chalara	24
bac2477	fungi220	-0.942857	negative	0.0166667	External	bac	2.2416667	g__Crenobacter	4	fungi	8.179143712	g__Sphaerosporella	13
bac2477	fungi280	-0.942857	negative	0.0166667	External	bac	2.2416667	g__Crenobacter	4	fungi	2.74970385	g__Verticillium	24
bac2477	fungi300	-0.942857	negative	0.0166667	External	bac	2.2416667	g__Crenobacter	4	fungi	4.37938565	g__Fusarium	13
bac2727	fungi305	-0.942857	negative	0.0166667	External	bac	7.4340264	g__Shewanella	3	fungi	2.434577802	g__Stylonectria	14
bac2727	fungi357	-0.942857	negative	0.0166667	External	bac	7.4340264	g__Shewanella	3	fungi	3.715935126	g__Xylaria	14
bac2849	fungi335	-0.942857	negative	0.0166667	External	bac	3.071906	g__Leclercia	1	fungi	4.879835406	g__Podospora	5
bac2857	fungi128	-0.942857	negative	0.0166667	External	bac	3.6971452	g__Raoultella	6	fungi	2.618927506	g__Rasamsonia	33
bac2857	fungi202	-0.942857	negative	0.0166667	External	bac	3.6971452	g__Raoultella	6	fungi	5.481009688	g__Pseudogymnoascus	11
bac2857	fungi330	-0.942857	negative	0.0166667	External	bac	3.6971452	g__Raoultella	6	fungi	4.362996709	g__Chaetomium	33
bac2857	fungi464	-0.942857	negative	0.0166667	External	bac	3.6971452	g__Raoultella	6	fungi	3.881790576	g__Hysterangium	33
bac2857	fungi516	-0.942857	negative	0.0166667	External	bac	3.6971452	g__Raoultella	6	fungi	5.101347038	g__Rhodotorula	33
bac2857	fungi523	-0.942857	negative	0.0166667	External	bac	3.6971452	g__Raoultella	6	fungi	5.735294062	g__Puccinia	33
bac2868	fungi335	-0.942857	negative	0.0166667	External	bac	6.8661079	g__Erwinia	1	fungi	4.879835406	g__Podospora	5
bac2888	fungi113	-0.942857	negative	0.0166667	External	bac	4.2211975	g__Xenorhabdus	12	fungi	2.768580701	g__Cladophialophora	52
bac2888	fungi128	-0.942857	negative	0.0166667	External	bac	4.2211975	g__Xenorhabdus	12	fungi	2.618927506	g__Rasamsonia	33
bac2888	fungi168	-0.942857	negative	0.0166667	External	bac	4.2211975	g__Xenorhabdus	12	fungi	2.893533421	g__Golovinomyces	23
bac2888	fungi172	-1	negative	0.0027778	External	bac	4.2211975	g__Xenorhabdus	12	fungi	3.218715812	g__Coleophoma	58
bac2888	fungi222	-1	negative	0.0027778	External	bac	4.2211975	g__Xenorhabdus	12	fungi	8.573166259	g__Wilcoxina	58
bac2888	fungi330	-0.942857	negative	0.0166667	External	bac	4.2211975	g__Xenorhabdus	12	fungi	4.362996709	g__Chaetomium	33
bac2888	fungi337	-1	negative	0.0027778	External	bac	4.2211975	g__Xenorhabdus	12	fungi	3.953337679	g__Thermothielavioides	58
bac2888	fungi358	-0.942857	negative	0.0166667	External	bac	4.2211975	g__Xenorhabdus	12	fungi	2.494774469	g__Monosporascus	23
bac2888	fungi359	-0.942857	negative	0.0166667	External	bac	4.2211975	g__Xenorhabdus	12	fungi	3.210722678	g__Phialemoniopsis	52
bac2888	fungi464	-0.942857	negative	0.0166667	External	bac	4.2211975	g__Xenorhabdus	12	fungi	3.881790576	g__Hysterangium	33
bac2888	fungi516	-0.942857	negative	0.0166667	External	bac	4.2211975	g__Xenorhabdus	12	fungi	5.101347038	g__Rhodotorula	33
bac2888	fungi523	-0.942857	negative	0.0166667	External	bac	4.2211975	g__Xenorhabdus	12	fungi	5.735294062	g__Puccinia	33
bac2893	fungi168	-0.942857	negative	0.0166667	External	bac	4.0609601	g__Dickeya	3	fungi	2.893533421	g__Golovinomyces	23
bac2893	fungi358	-0.942857	negative	0.0166667	External	bac	4.0609601	g__Dickeya	3	fungi	2.494774469	g__Monosporascus	23
bac2899	fungi54	-0.942857	negative	0.0166667	External	bac	7.1060224	g__Yersiniaceae_Unclassified	15	fungi	2.012232792	g__Pyrenochaeta	11
bac2899	fungi128	-1	negative	0.0027778	External	bac	7.1060224	g__Yersiniaceae_Unclassified	15	fungi	2.618927506	g__Rasamsonia	33
bac2899	fungi172	-0.942857	negative	0.0166667	External	bac	7.1060224	g__Yersiniaceae_Unclassified	15	fungi	3.218715812	g__Coleophoma	58
bac2899	fungi181	-0.942857	negative	0.0166667	External	bac	7.1060224	g__Yersiniaceae_Unclassified	15	fungi	3.915058394	g__Lachnellula	34
bac2899	fungi188	-0.942857	negative	0.0166667	External	bac	7.1060224	g__Yersiniaceae_Unclassified	15	fungi	3.46213568	g__Botrytis	34
bac2899	fungi222	-0.942857	negative	0.0166667	External	bac	7.1060224	g__Yersiniaceae_Unclassified	15	fungi	8.573166259	g__Wilcoxina	58
bac2899	fungi330	-1	negative	0.0027778	External	bac	7.1060224	g__Yersiniaceae_Unclassified	15	fungi	4.362996709	g__Chaetomium	33
bac2899	fungi337	-0.942857	negative	0.0166667	External	bac	7.1060224	g__Yersiniaceae_Unclassified	15	fungi	3.953337679	g__Thermothielavioides	58
bac2899	fungi464	-1	negative	0.0027778	External	bac	7.1060224	g__Yersiniaceae_Unclassified	15	fungi	3.881790576	g__Hysterangium	33
bac2899	fungi516	-1	negative	0.0027778	External	bac	7.1060224	g__Yersiniaceae_Unclassified	15	fungi	5.101347038	g__Rhodotorula	33
bac2899	fungi523	-1	negative	0.0027778	External	bac	7.1060224	g__Yersiniaceae_Unclassified	15	fungi	5.735294062	g__Puccinia	33
bac2899	fungi526	-0.942857	negative	0.0166667	External	bac	7.1060224	g__Yersiniaceae_Unclassified	15	fungi	9.289104509	g__Phaffia	10
bac2899	fungi531	-0.942857	negative	0.0166667	External	bac	7.1060224	g__Yersiniaceae_Unclassified	15	fungi	6.060552081	g__Cryptococcus	11
bac2899	fungi538	-0.942857	negative	0.0166667	External	bac	7.1060224	g__Yersiniaceae_Unclassified	15	fungi	4.446936181	g__Tremellales_norank	10
bac2903	fungi54	-0.942857	negative	0.0166667	External	bac	5.6848137	g__Ewingella	15	fungi	2.012232792	g__Pyrenochaeta	11
bac2903	fungi128	-1	negative	0.0027778	External	bac	5.6848137	g__Ewingella	15	fungi	2.618927506	g__Rasamsonia	33
bac2903	fungi172	-0.942857	negative	0.0166667	External	bac	5.6848137	g__Ewingella	15	fungi	3.218715812	g__Coleophoma	58
bac2903	fungi181	-0.942857	negative	0.0166667	External	bac	5.6848137	g__Ewingella	15	fungi	3.915058394	g__Lachnellula	34
bac2903	fungi188	-0.942857	negative	0.0166667	External	bac	5.6848137	g__Ewingella	15	fungi	3.46213568	g__Botrytis	34
bac2903	fungi222	-0.942857	negative	0.0166667	External	bac	5.6848137	g__Ewingella	15	fungi	8.573166259	g__Wilcoxina	58
bac2903	fungi330	-1	negative	0.0027778	External	bac	5.6848137	g__Ewingella	15	fungi	4.362996709	g__Chaetomium	33
bac2903	fungi337	-0.942857	negative	0.0166667	External	bac	5.6848137	g__Ewingella	15	fungi	3.953337679	g__Thermothielavioides	58
bac2903	fungi464	-1	negative	0.0027778	External	bac	5.6848137	g__Ewingella	15	fungi	3.881790576	g__Hysterangium	33
bac2903	fungi516	-1	negative	0.0027778	External	bac	5.6848137	g__Ewingella	15	fungi	5.101347038	g__Rhodotorula	33
bac2903	fungi523	-1	negative	0.0027778	External	bac	5.6848137	g__Ewingella	15	fungi	5.735294062	g__Puccinia	33
bac2903	fungi526	-0.942857	negative	0.0166667	External	bac	5.6848137	g__Ewingella	15	fungi	9.289104509	g__Phaffia	10
bac2903	fungi531	-0.942857	negative	0.0166667	External	bac	5.6848137	g__Ewingella	15	fungi	6.060552081	g__Cryptococcus	11
bac2903	fungi538	-0.942857	negative	0.0166667	External	bac	5.6848137	g__Ewingella	15	fungi	4.446936181	g__Tremellales_norank	10
bac2907	fungi600	-0.942857	negative	0.0166667	External	bac	6.0492074	g__Rouxiella	1	fungi	1.616016228	g__Parasitella	7
bac2908	fungi202	-0.942857	negative	0.0166667	External	bac	7.6231609	g__Serratia	1	fungi	5.481009688	g__Pseudogymnoascus	11
bac2909	fungi600	-0.942857	negative	0.0166667	External	bac	9.7438463	g__Yersinia	1	fungi	1.616016228	g__Parasitella	7
bac2911	fungi309	-0.942857	negative	0.0166667	External	bac	3.3088262	g__Plesiomonas	1	fungi	3.218675805	g__Purpureocillium	2
bac2936	fungi305	-0.942857	negative	0.0166667	External	bac	2.3622681	g__Methylomonas	3	fungi	2.434577802	g__Stylonectria	14
bac2936	fungi357	-0.942857	negative	0.0166667	External	bac	2.3622681	g__Methylomonas	3	fungi	3.715935126	g__Xylaria	14
bac2954	fungi600	-0.942857	negative	0.0166667	External	bac	4.6375309	g__Psychrobacter	2	fungi	1.616016228	g__Parasitella	7
bac2984	fungi320	-0.942857	negative	0.0166667	External	bac	4.4012654	g__Halomonas	3	fungi	2.830798666	g__Scedosporium	4
bac2985	fungi58	-0.942857	negative	0.0166667	External	bac	3.7449998	g__Halotalea	4	fungi	3.013375709	g__Didymella	3
bac2985	fungi220	-0.942857	negative	0.0166667	External	bac	3.7449998	g__Halotalea	4	fungi	8.179143712	g__Sphaerosporella	13

bac2985	fungi300	-0.942857	negative	0.0166667	External	bac	3.7449998	g__Halotalea	4	fungi	4.37938565	g__Fusarium	13
bac3057	fungi335	-0.942857	negative	0.0166667	External	bac	3.8726999	g__Marinobacter	1	fungi	4.879835406	g__Podospora	5
pro155	fungi128	-0.942857	negative	0.0166667	External	pro	4.55661	g__Onchocerca	20	fungi	2.618927506	g__Rasamsonia	33
pro155	fungi134	-0.942857	negative	0.0166667	External	pro	4.55661	g__Onchocerca	20	fungi	3.214064268	g__Emmonsia	3
pro155	fungi330	-0.942857	negative	0.0166667	External	pro	4.55661	g__Onchocerca	20	fungi	4.362996709	g__Chaetomium	33
pro155	fungi464	-0.942857	negative	0.0166667	External	pro	4.55661	g__Onchocerca	20	fungi	3.881790576	g__Hysterangium	33
pro155	fungi472	-0.942857	negative	0.0166667	External	pro	4.55661	g__Onchocerca	20	fungi	4.799642621	g__Grifola	2
pro155	fungi516	-0.942857	negative	0.0166667	External	pro	4.55661	g__Onchocerca	20	fungi	5.101347038	g__Rhodotorula	33
pro155	fungi523	-0.942857	negative	0.0166667	External	pro	4.55661	g__Onchocerca	20	fungi	5.735294062	g__Puccinia	33
pro155	fungi526	-1	negative	0.0027778	External	pro	4.55661	g__Onchocerca	20	fungi	9.289104509	g__Phaffia	10
pro155	fungi528	-0.942857	negative	0.0166667	External	pro	4.55661	g__Onchocerca	20	fungi	5.191116269	g__Naganishia	2
pro155	fungi532	-0.942857	negative	0.0166667	External	pro	4.55661	g__Onchocerca	20	fungi	5.783314283	g__Kwoniella	2
pro155	fungi538	-1	negative	0.0027778	External	pro	4.55661	g__Onchocerca	20	fungi	4.446936181	g__Tremellales_norank	10
virus30	pro141	-0.942857	negative	0.0166667	External	virus	8.2318864	f__Myoviridae	4	pro	3.427027558	g__Emiliania	5
virus34	pro2	-0.942857	negative	0.0166667	External	virus	6.4627892	f__Schitoviridae	4	pro	4.58450819	g__Plasmodium	11
bac68	pro153	-1	negative	0.0027778	External	bac	2.3785271	g__Dietzia	1	pro	6.078485924	g__Brugia	12
bac108	pro155	-1	negative	0.0027778	External	bac	0.585562	g__Pseudokineococcus	12	pro	4.556609995	g__Onchocerca	20
bac172	pro155	-0.942857	negative	0.0166667	External	bac	2.4223218	g__Alpinimonas	15	pro	4.556609995	g__Onchocerca	20
bac194	pro153	-0.927634	negative	0.0076658	External	bac	2.4508327	g__Gulosibacter	2	pro	6.078485924	g__Brugia	12
bac202	pro155	-0.942857	negative	0.0166667	External	bac	8.2918427	g__Leifsonia	15	pro	4.556609995	g__Onchocerca	20
bac210	pro155	-0.942857	negative	0.0166667	External	bac	6.3490883	g__Mycetocola	15	pro	4.556609995	g__Onchocerca	20
bac806	pro2	-0.942857	negative	0.0166667	External	bac	4.7640354	g__Elizabethkingia	2	pro	4.58450819	g__Plasmodium	11
bac806	pro178	-1	negative	0.0027778	External	bac	4.7640354	g__Elizabethkingia	2	pro	3.14402296	g__Trichuris	17
bac834	pro178	-0.942857	negative	0.0166667	External	bac	11.532559	g__Pedobacter	1	pro	3.14402296	g__Trichuris	17
bac1164	pro155	-0.942857	negative	0.0166667	External	bac	4.1749563	g__Filibacter	15	pro	4.556609995	g__Onchocerca	20
bac1175	pro155	-0.942857	negative	0.0166667	External	bac	8.1578089	g__Sporosarcina	15	pro	4.556609995	g__Onchocerca	20
bac2320	pro2	-0.942857	negative	0.0166667	External	bac	3.8689276	g__Candidimonas	2	pro	4.58450819	g__Plasmodium	11
bac2320	pro178	-1	negative	0.0027778	External	bac	3.8689276	g__Candidimonas	2	pro	3.14402296	g__Trichuris	17
bac2334	pro178	-0.942857	negative	0.0166667	External	bac	7.0433843	g__Pigmentiphaga	1	pro	3.14402296	g__Trichuris	17
bac2352	pro2	-0.942857	negative	0.0166667	External	bac	4.9096356	g__Pandoraea	2	pro	4.58450819	g__Plasmodium	11
bac2352	pro178	-1	negative	0.0027778	External	bac	4.9096356	g__Pandoraea	2	pro	3.14402296	g__Trichuris	17
bac2488	pro178	-0.942857	negative	0.0166667	External	bac	4.1961939	g__Candidatus Nitrotoga	1	pro	3.14402296	g__Trichuris	17
bac2727	pro178	-0.942857	negative	0.0166667	External	bac	7.4340264	g__Shewanella	3	pro	3.14402296	g__Trichuris	17
bac2824	pro2	-1	negative	0.0027778	External	bac	4.2634402	g__Sodalis	3	pro	4.58450819	g__Plasmodium	11
bac2824	pro178	-0.942857	negative	0.0166667	External	bac	4.2634402	g__Sodalis	3	pro	3.14402296	g__Trichuris	17
bac2887	pro2	-0.942857	negative	0.0166667	External	bac	6.4197995	g__Providencia	1	pro	4.58450819	g__Plasmodium	11
bac2893	pro125	-0.942857	negative	0.0166667	External	bac	4.0609601	g__Dickeya	3	pro	1.222598268	g__Bodo	2
bac2936	pro178	-0.942857	negative	0.0166667	External	bac	2.3622681	g__Methylomonas	3	pro	3.14402296	g__Trichuris	17
bac2984	pro2	-0.942857	negative	0.0166667	External	bac	4.4012654	g__Halomonas	3	pro	4.58450819	g__Plasmodium	11
bac2985	pro159	-0.942857	negative	0.0166667	External	bac	3.7449998	g__Halotalea	4	pro	3.51820975	g__Diploscapter	4

```
library(psych)
data1 <- read.table(Args[3],header=T,sep="\t",check.names = FALSE,row.names = 1)
data2 <- read.table(Args[4],header=T,sep="\t",check.names = FALSE,row.names = 1)
data1 <- data.frame(t(data1))
data2 <- data.frame(t(data2))
cordata <- corr.test(data1,data2,method = "spearman",adjust="none")
#cordata <- corr.test(data1,data2,method = "spearman")
write.table(data.frame(cordata$r),file =
paste(Args[3],".",Args[4],".cor.txt",sep=""),sep="\t",quote=F,row.names = T)
write.table(data.frame(cordata$p),file =
paste(Args[3],".",Args[4],".p.txt",sep=""),sep="\t",quote=F,row.names = T)
```

```
R --no-save < psych.cor.r data1.txt data2.txt
```
